# The Catalase Gene Family in Cotton: Genome-Wide Characterization and Bioinformatics Analysis

**DOI:** 10.3390/cells8020086

**Published:** 2019-01-24

**Authors:** Wei Wang, Yingying Cheng, Dongdong Chen, Dan Liu, Mengjiao Hu, Jie Dong, Xiaopei Zhang, Lirong Song, Fafu Shen

**Affiliations:** State Key Laboratory of Crop Biology, College of Agronomy, Shandong Agricultural University, NO. 61 Daizong Street, Tai’an 271018, China; weiwang@sdau.edu.cn (W.W.); 17661212080@163.com (Y.C.); chendd@sdau.edu.cn (D.C.); zkybliudan@163.com (D.L.); 15664448869@163.com (M.H.); dongjie345345@163.com (J.D.); xpzhang0108@163.com (X.Z.); 18865381836@163.com (L.S.)

**Keywords:** catalase, reactive oxygen species, cotton, Verticillium wilt, expression and regulation

## Abstract

Catalases (CATs), which were coded by the catalase gene family, were a type notably distinguished ROS-metabolizing proteins implicated to perform various physiological functions in plant growth, development and stress responses. However, no systematical study has been performed in cotton. In the present study, we identified 7 and 7 *CAT* genes in the genome of *Gossypium hirsutum* L. Additionally, *G. barbadense* L., respectively. The results of the phylogenetic and synteny analysis showed that the *CAT* genes were divided into two groups, and whole-genome duplication (WGD) or polyploidy events contributed to the expansion of the *Gossypium* *CAT* gene family. Expression patterns analysis showed that the *CAT* gene family possessed temporal and spatial specificity and was induced by the *Verticillium dahliae* infection. In addition, we predicted the putative molecular regulatory mechanisms of the *CAT* gene family. Based on the analysis and preliminary verification results, we hypothesized that the *CAT* gene family, which might be regulated by transcription factors (TFs), alternative splicing (AS) events and miRNAs at different levels, played roles in cotton development and stress tolerance through modulating the reactive oxygen species (ROS) metabolism. This is the first report on the genome-scale analysis of the cotton *CAT* gene family, and these data will help further study the roles of *CAT* genes during stress responses, leading to crop improvement.

## 1. Introduction

Reactive oxygen species (ROS), such as superoxide anion (O_2_^−^), hydrogen peroxide (H_2_O_2_), hydroxyl radical (·OH), singlet oxygen (^1^O_2_), are formed as a toxic byproduct of the normal metabolism of oxygen (O_2_) and are also considered to play a crucial role in plant biology as signaling molecules [1]. Recent several studies have revealed that ROS are essential for maintaining normal cellular functions, as opposed to having a toxic effect on cells [2]. Even cell death that was previously considered a result from oxidative damage is now thought to be a ROS-mediated physiological pathway for programmed cell death, rather than directly killing the cells [3,4]. Additionally, ROS levels that are too low are cytostatic for cells, whereas ROS levels that are too high are cytotoxic [5]. Thus, maintaining a basal level of ROS is essential for proper cellular ROS signaling, and this level is achieved by the fine balance between ROS production and ROS scavenging [6]. The major ROS-scavenging pathways of plants include superoxide dismutases (SODs), found in almost all cellular compartments, the water-water cycle in chloroplasts; the ascorbate-glutathione cycle in chloroplasts, cytosol, mitochondria, apoplast and peroxisomes; glutathione peroxidases (GPXs) cycle in cytosol; and catalases (CATs) in peroxisomes [7,8]. Among these ROS-metabolizing proteins, CATs, which are indispensable for ROS detoxification during stress, are highly active enzymes that do not require cellular reductants as they primarily catalyze a dismutase reaction [9,10].

CATs, which can convert H_2_O_2_ into water (H_2_O) and O_2_, is an enzyme found in nearly all living organisms. In animals, CATs are encoded by a unique gene. In plants, CATs are encoded by a small multi-gene family and have been reported in different species. For example, there are three gene members in *Arabidopsis thaliana* (L.) Heynh. [11], tobacco (*Nicotiana plumbaginifolia* Viviani) [12], maize (*Zea mays* L.) [13], rice (*Oryza sativa* L.) [14] and pumpkin (*Cucurbita* Linn.) [15]; 4 in cucumber (*Cucumis sativus* L.) [16]; 2 in common barley (*Hordeum vulgare* L.) [17]; and 1 in sweet potato (*Ipomoea batatas* (L.) Poir.) [18], castor bean (*Ricinus communis* L.) [19] and tomato (*Lycopersicon esculentum* Mill.) [20].

Many studies have suggested that the gene expression of plant CATs is regulated temporally and spatially, is involved in regulating growth and development and responds to environmental stimuli [9,18,21,22,23]. In *Arabidopsis*, CAT1 is an important H_2_O_2_ scavenger that is generated under various abiotic stresses. CAT2 and CAT3 are major players in the removal of H_2_O_2_ contributed to ROS homeostasis in light or darkness, respectively. Additionally, CAT2 and CAT3 are the major isoforms in *Arabidopsis* rosette tissue [10]. Besides, CAT2 is induced under cold and drought stresses, and CAT3 is mainly activated by abscisic acid and oxidative treatments as well as at the senescence stage [11]. Studying catalase mutants and knockdowns in *Arabidopsis*, it was confirmed that *CAT2* encodes the major leaf catalase isoform and that the function of this enzyme is closely linked to photorespiration [24]. The *Arabidopsis* mutants of *CAT2* typically display patches of chlorosis and necrotic lesions [25]. In sweet potato, the expression of *CAT1* is induced by ethephon and regulated by reduced glutathione, the NADPH oxidase inhibitor diphenylene iodonium (DPI), the calcium ion chelator EGTA and cycloheximide [18]. In tobacco, *CAT1* and *CAT2* are detected in non-senescent leaves; the expression level of *CAT2*, but not *CAT1*, is significantly decreased in senescent leaves compared to non-senescent leaves. *CAT3* is detected in both non-senescent and senescing leaves [26]. Transgenic tobacco plants expressing the maize *CAT2* gene have enhanced the resistance to pathogen infection [27]. In rice, the expression of *CatA* and *CatC* is controlled by circadian rhythm, with a morning-specific phase for *CatC* and an evening-specific phase for *CatA*. Additionally, the over-expressed *CatA* and *CatC*, but not over-expressed *CatB*, improved drought stress tolerance in transgenic rice. However, over-expressed *CatB* exhibited root growth inhibition [14]. Under salt stress, CatC interacts with the salt tolerance receptor-like cytoplasmic kinase 1 (STRK1) via palmitoylation, and CatC is phosphorylated mainly at Tyr210 and activated by STRK1, thereby regulating H_2_O_2_ homeostasis and improving the salt tolerance in rice [9]. These data, which were displayed as a table (Table S1), have suggested that some important biological processes are always related with the transcription of *CAT* genes, and subsequently alter the enzyme activity of CAT, leading to redox homeostasis in plant cells. However, tissue- and/or stress-specific expression profiles and their regulation of *CAT* genes are still largely unknown in cotton.

Cotton (*Gossypium* spp.), among which only four species, including two allotetraploids (*Gossypium hirsutum* L. and *G. barbadense* L.) and two diploids (*G. herbaceum* L. and *G. arboreum* L.), producing spannable fiber, is a widely cultivated polyploid crop and provides fiber, seed oil, and protein meal [28]. Among the two cultivated allotetraploids, the upland cotton *G. hirsutum* L. is characterized by its high yield yet moderate fiber qualities, whereas the sea-island cotton *G. barbadense* L. accounts for nearly 3% of the world’s cotton production and is famous for its superior quality fibers, based on the length, strength and fineness of its fibers [29]. Although they have a common ancestor [30], upland cotton and sea-island cotton have diverged with regards to their resistance to Verticillium wilt (VW), exhibiting generally susceptible and strongly resistant characteristics, respectively [29]. VW is one of the most important diseases in cotton and caused by the soil-borne fungal pathogen *Verticillium dahliae* Kleb., which invades over 350 species of eudicot plant hosts worldwide [31]. VW will lead to chlorosis and the wilting of leaves or defoliation, the discoloration of vascular tissues and, ultimately, even death and is especially destructive in upland cotton [31]. Several studies of the defense responses to VW in cotton have demonstrated that the rapid production of ROS (also termed ROS burst) occurs in the apoplast during the recognition of pathogen infection. The ROS burst is considered an important component of the early plant immune response. Accumulating evidence has suggested that many VW-related genes, such as *GbWARKY1*, *GbERF*, *GbNRX1*, *GbEDS1*, *GhSSN*, *GhMLP28*, *GhNDR1*, *GhMKK2* and *GhBAK1*, play a crucial role in the homeostasis of apoplastic ROS in response to *V. dahliae* Kleb. infection in cotton [32,33,34,35,36]. Recently, the regulation mechanism of CAT-mediated ROS homeostasis has been revealed when rice is subjected to salt stress [9]. However, among plants, especially in cotton, the role and molecular regulatory system of CATs that are responsible for ROS homeostasis during the immune response of VW, is not well understood.

The release of different cotton whole-genome sequence data, including *Gossypium arboreum* L. [37], *Gossypium raimondii* Ulbr. [38], *Gossypium hirsutum* L. [39] and *Gossypium barbadense* L. [40], has made it possible to systematically identify and analyze the cotton *CAT* genes on a genome-scale level, and has thus enriched our understanding on their role and molecular regulatory system in VW resistance in cotton. In this research, we systematically identify *CAT* genes in two sequenced allotetraploids cotton species for the first time, and analyze their genomic organization, gene structure and phylogenetic relationships. Subsequently, the expression patterns of the *CAT* gene family in response to *V. dahliae* Kleb. infection is analyzed. Finally, *cis*-elements in the putative promoters of *CAT* are analyzed, and miRNA target sites of *CAT* are predicted. Our study provided a comprehensive analysis of the *Gossypium CAT* gene family and clarified the important roles of the CAT genes in VW resistance, and provided important information for further exploring the functional differences between two allotetraploid cotton species.

## 2. Materials and Methods

### 2.1. Databases

The four cotton genome files (*Gossypium arboreum* L., BGI; *Gossypium raimondii* Ulbr., JGI; *Gossypium hirsutum* L., NAU; *Gossypium barbadense* L., NAU) were downloaded from the Cotton Functional Genomics Database (CottonFGD) (https://cottonfgd.org/) [41]. The four other plant genome files (*Arabidopsis thaliana* (Linn.) Heynh.; *Glycine max* (Linn.) Merr.; *Theobroma cacao* L.; *Vitis vinifera* L.) were obtained from the JGI database (http://www.phytozome.net).

### 2.2. Sequence Identification and Functional Annotation Analysis

The *CAT* genes in the genomes of four kinds of cotton types and the other four plants were identified as described in our previous study [8]. Briefly, the HMMER 3.1 (http://www.hmmer.org/) in the default parameter settings and BLAST algorithm for Proteins (BLASTP) with the threshold expectation value set to 0.0001 were performed using the Hidden Markov Model (HMM) (version 3.0) profiles of the Catalase (PF00199) and Catalase-related immune-responsive domain (PF06628) obtained from the Pfam database (http://pfam.xfam.org/) as the query. Then, we merged all hits obtained using HMM and BLASTP searches and removed the redundant hits. All non-redundant protein sequences were retrieved and further analyzed with SMART (Simple Modular Architecture Research Tool) (version 8.0) (http://smart.embl-heidelberg.de) and InterProScan (version 4.8) (http://www.ebi.ac.uk/interpro/) to examine the inclusion of the conserved domain of the CAT protein in each candidate sequence. Finally, the identified candidate *CAT* genes were further confirmed manually to eliminate spurious sequences.

The details of upland cotton and sea-island cotton *CAT* genes, including gene features, transcript features and protein statistics, were collected from CottonFGD (https://cottonfgd.org/). The exons-intron structures of *CAT* genes were graphically visualized by the Gene Structure Draw Server (GSDraw) web server (http://www.bioinfogenome.net/piece/). Predictions of subcellular localizations and Gene Ontology (GO) were performed with the PredictProtein web server (https://open.predictprotein.org/). The Gene Ontology (GO) annotation was performed with the Gene Ontology Consortium web server (http://geneontology.org/) using *A. thaliana* as the reference species, based on their molecular functions, biological processes, and cellular localizations. The results of the GO annotation were visualized and plotted by the Web Gene Ontology Annotation Plot (WEGO, version 2.0) (http://wego.genomics.org.cn/). The conserved domains of all the protein sequences encoded by candidate *CAT* genes were presented by the IBS software (version 1.0.3) (http://ibs.biocuckoo.org/).

### 2.3. Phylogenetic and Synteny Analysis

The full-length amino acid sequence encoded by *CAT* genes were aligned with the ClustalW program (version 2.0) with the default settings, and then manually adjusted in MEGA6.06 [42]. Subsequently, we constructed the neighbor-joining (NJ) tree with 1000 bootstrap replicates using the Jones-Taylor-Thornton (JTT) substitution model in MEGA 6.06 with a cut-off value of 60% for the condensed tree. Additionally, the phylogenetic tree of *Gossypium* and four other genomes descended from common eudicot genome ancestor in Rosids used in the study was gathered from the PLAZA database (version 4.0) [43]. MCScanX, a package developed by the Plant Genome Duplication Database (PGDD) (http://chibba.pgml.uga.edu/duplication/), was used to perform a synteny examination of paralogous genes among the genomes of the four cotton species said above based on a previously described method [8]. The results of the synteny analysis were visualized with Circos-0.69 (http://circos.ca/).

### 2.4. Transcription Factor Binding Sites Prediction

The genomic sequences of 1.5 kb upstream of the translation start site (TSS) of each *CAT* gene was extracted from the assembly files of *G. hirsutum* L. and *G. barbadense* L. downloaded from CottonFGD (https://cottonfgd.org/). The putative transcription factor (TF) binding sites (TFBSs) of the *CAT* gene promoter regions were predicted using the Binding Site Prediction tool in the PlantTFDB 4.0 serve, a central hub for TF and regulatory interactions in plants (http://planttfdb.cbi.pku.edu.cn/), with a stricter parameter: threshold *p*-value ≤ 1 × 10^−6^. Using the sets of high-quality, non-redundant binding motifs of TFs for 156 species with whole genome sequences, the putative TFBSs of the *CAT* gene promoter regions were scanned.

### 2.5. Potential Alternative Splicing Events Analysis

We gathered all potential isoforms of the upland cotton and sea-island cotton *CAT* gene family from CottonFGD (https://cottonfgd.org/) with the JBrowse tool. Transcript structures of each isoform were graphically visualized by the Gene Structure Display Server (GSDS) (version 2.0) (http://gsds.cbi.pku.edu.cn/). Alternative splicing (AS) events were identified from transcript structures of each isoform and classified into intron retention (IR), exon skipping (ES), alternative 5′ donor sites (AD) and alternative 3′ acceptor sites (AA).

### 2.6. Putative microRNA Target Sites Analysis

We obtained microRNA (miRNA) sequences of upland cotton and sea-island cotton from miRBase (http://www.mirbase.org/), the Plant MicroRNA database (http://bioinformatics.cau.edu.cn/PMRD/), the Cotton EST database (http://www.leonxie.com/) and published articles [44,45,46,47,48,49,50,51,52]. *CAT* genes targeted by miRNAs were predicted by searching their coding sequence (CDS) regions for complementary sequences using the psRNATarget server with default parameters, except maximum expectation (E) = 4.0 (http://plantgrn.noble.org/psRNATarget/home). We selected the targeted sites with high degrees of complementarity shown in the Figure.

### 2.7. Plant Growth Conditions and Fungal Pathogen Infection Assays

Seeds of cotton (*G. hirsutum* L. cv. SF06 and *G*. *barbadense* L. cv. Hai7124) were surface-disinfected in 0.5% sodium hypochlorite (NaClO) for 5 min, and then washed five times with sterile distilled water. After they were transferred to sterile distilled water soaked sterile gauzes in Petri dishes (90-mm in diameter), they were placed in a biochemical incubator at room temperature (RT) for 48 h until they germinated. Any seeds presenting internal fungal contamination were discarded. Seedlings of similar size were selected and sown on commercial sterilized soil at 25/23 °C (day/night), with a 16 h light/8 h dark schedule for 2 weeks.

Fungal pathogen inoculations were performed using *V. dahliae* (strain Vd414, a highly toxic and defoliant strain). The strain was cultivated in a Potato Dextrose Agar (PDA) plate at 25 °C for one week from storage at –80 °C, and then high activity hyphae were collected and then cultivated in Potato Dextrose Broth (PDB) medium at 25 °C for 3–5 days. The suspension liquid was filtered through four layers of gauze (to remove mycelia), and the conidial concentration was adjusted to approximately 10^7^ spores per ml with sterile distilled water for inoculation.

The two-week-old seedlings of the susceptible cultivar SF06 of *G. hirsutum* L. Additionally, the resistant cultivar Hai7124 of *G*. *barbadense* L. were inoculated with 10^7^ spores per ml of Vd414 using the root dip method. Seedlings were gently uprooted, rinsed in sterile water, inoculated into a spore suspension for 50 min, and then the inoculated plants were returned to new pots containing sterilized soil and incubated at 25 °C under a 16 h photoperiod. Six individual seedling roots were collected at 0, 6, 12 and 24 h post inoculation. Control plants were treated with sterile distilled water in the same way, and all roots samples were immediately frozen in liquid nitrogen and stored at –80 °C until total RNA extraction.

### 2.8. RNA Isolation and Expression Profiling Analysis

Total RNA from 100 mg of plant samples was isolated using the RNAprep Pure Plant Kit (Polysaccharides & Polyphenolics-rich, DP441) (TIANGEN, Beijing, China). The concentrations of the isolated RNA samples were determined by 1.5% agarose gel electrophoresis and a NanoDrop 2000 Spectrophotometer (Thermo Fisher Scientific, Wilmington, DE, USA). Additionally, the RNA samples (1 μg per reaction) were reversely transcribed into complementary DNA (cDNA) using the Mir-X™ miRNA First-Strand Synthesis Kit (TaKaRa, Dalian, China). The reverse transcription polymerase chain reaction (RT-PCR) was performed to validate the accuracy of alternative splicing (AS) events. The following program was used for RT-PCR: 50 °C for 30 min, 94 °C for 2 min followed by 30 cycles at 94 °C for 30 s, 60 °C for 30 s and 72 °C for 30 s, followed by a 5 min extension step at 72 °C. The PCR products were separated on agarose gel electrophoresis and quantified using a Gel Imaging and Analysis System (Beijing Sage, China). Quantitative polymerase chain reaction (qPCR) was performed using a QuantStudio™ 6 Flex Real-Time PCR System (Applied Biosystems™, Carlsbad, CA, USA) and SYBR^®^ Premix Ex Taq™II (Tli RNaseH Plus) (RR820A) (TaKaRa, Dalian, China) with three biological replicates and three technical replicates in a 20-μL reaction volume, which contained 1.6 μL of diluted cDNA template. PCRs included an initial denaturation at 95 °C for 3 min, followed by 40 cycles at 95 °C for 10 s, 60 °C for 20 s, and 72 °C for 30 s in a 96-well plate. Following the PCR, a melting curve analysis was performed. The cycle threshold (C_t_) was used for the relative quantification of the input target number. Cotton ubiquitin 7 (*UBQ7*) was used as an endogenous control. Relative fold difference represents the number of treated target gene transcript copies relative to the number of untreated gene transcript copies and was calculated according to the 2^−ΔΔCT^ method [8]. All gene-specific primers or miRNA-specific 5′-primers were gathered from the qPrimerDB database (http://biodb.swu.edu.cn/qprimerdb) [53], the ICG database (http://icg.big.ac.cn/index.php/Main_Page) [54], or designed using Primer Premier 5.0 and checked by the Blast tool (Table S2).

The expression value (FPKMs, fragments per kilobase per million reads) of cotton, which was calculated from high-throughput RNA-sequencing data and gathered from ccNET (http://structuralbiology.cau.edu.cn/gossypium/) and CottonFGD (https://cottonfgd.org/), was used to systematically analyze the expression profiling of cotton *CAT* genes in different tissues and under different stresses. Gene expression levels were calculated according to the log_2_ of FPKMs values and the default empirical abundance threshold of FPKM > 1 was used to identify the expressed gene. The heat maps were plotted by using the Cluster 3.0 software and TreeView (version 3.0).

### 2.9. Gene Cloning, Vector Construction, Genetic Transformation

Based on the known sequences of two allotetraploid cotton species, the gene-specific primers were designed using Primer Premier 5.0 to amplify the homologous genes of *CAT* with complete open reading frames (ORFs) in *G. hirsutum* L. cv. SF06 and *G. barbadense* L. cv. Hai7124. Phanta^®^ Max Super-Fidelity DNA Polymerase (Vazyme, Nanjing, China) was used in standard PCR reactions from cotton cDNA. The PCR product was ligated into the *pEASY*^®^-Blunt (TransGen, Beijing, China) to generate cloning vectors transformed into bacterial strains of *E. coli* DH5α. At least ten clones per gene were randomly selected to sequence.

## 3. Results

### 3.1. Identification of CAT Genes in Upland Cotton and Sea-Island Cotton

We used HMMER 3.1 and BLASTP to search for *CAT* genes in the two released allotetraploid cotton genomes with the HMM profiles of the Catalase (PF00199) and Catalase-related immune-responsive domain (PF06628) obtained from the Pfam database as the queries. Then, we used the SMART and InterProScan programs to verify the predicted sequences. A total of 7 and 7 putative *CAT* genes were identified in *G. hirsutum* L. and *G. barbadense* L., respectively. The gene names, locus IDs and other features were shown in Table 1.

### 3.2. Sequence Analysis of GhCATs and GbCATs

**Figure 1:**
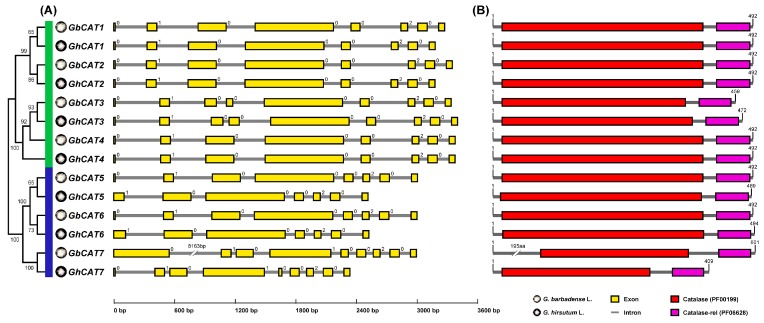
The sequence analysis of two allotetraploid cotton *CAT* genes. (**A**) Exon–intron structures. Yellow boxes and grey horizontal lines indicated exons and introns, respectively. Intron phases were shown by 0, 1 and 2. The *CATs* of two allotetraploid cotton clustered into two groups. Group I and II represented by green and blue, respectively. (**B**) Conserved domain compositions. Catalase core domain (*Catalase*, PF00199) and catalase-related immune-responsive domain (*Catalase-rel*, PF06628) were shown in the red and purple boxes, respectively.

Firstly, we collected the gene structure information of *GhCATs* and *GbCATs* in the gene annotation file and visualized it using the web tool (Figure 1A). The *CAT* genes of upland cotton and sea-island cotton clustering into the same group showed similar gene structures. The result of the *GhCAT* gene structure revealed that the numbers of exons varied between seven and nine, with the lowest numbers of exons in *GhCAT5* and *GhCAT6*, and the highest numbers in *GhCAT3* and *GhCAT7*. The result of the *GbCAT* gene structure revealed that the numbers of exons varied between eight and nine, with 8 exons in *GbCAT1*, *GbCAT2*, *GbCAT4*, *GbCAT5* and *GbCAT6*, and 9 exons in *GbCAT3* and *GbCAT7*.

Subsequently, the conserved domain of candidate GhCAT and GbCAT protein sequences were analyzed (Figure 1B). Based on the domain analysis, all conceptual GhCAT and GbCAT proteins contained one catalase core domain (PF00199, *Catalase*) and one catalase immune-responsive domain (PF06628, *Catalase-rel*). *Catalase* was the fundamental domain in catalases. *Catalase-rel* domain was an immune-responsive amphipathic octa-peptide that was found in the C-terminal of catalases. Then, we examined the chromosomal locations of *GhCATs* and *GbCATs* (Table 1). There were 7 chromosomes/scaffolds (upland cotton: chromosomes A1, A2, A5, A7, D1, D3, D5; sea-island cotton: chromosomes A1, A2, A5, A7, D1, D5, scaffold_0637), respectively, all harboring 7 *CAT* genes. Additionally, the computational prediction of protein localization indicated that all GhCATs and GbCATs were localized in peroxisome (Table 1).

In addition, we noticed that there are great differences in the length of the genomic sequence and coding sequence, and the size of the first exon, between *GhCAT7* and *GbCAT7*, which was a pair of orthology genes. The first intron of *GbCAT7* reached 8163 bp, and the first exon of *GbCAT7* encoded 195 amino acids, which did not match with the conserved domains of classical catalases (Table 1 and Figure 1). Then, we found that the RNA-seq Junction Support value of the *GbCAT7*’s first intron was 0 from the Cotton Functional Genomics Database. The RNA-seq Junction Support value was the number of RNA-seq reads supporting each intron in the transcript structure page, and the larger the number, the more supporting reads and the more credible this gene structure was. Thus, we hypothesized that the gene structure of *GbCAT7* was annotated wrong because there were some erroneous assemblies when sequencing the whole genomes of sea-island cotton. To test this, the experimental investigations were carried out. Firstly, we aligned the *GbCAT7* CDS in the upland cotton genome database (cDNA, *G. hirsutum* (AD1), NAU) using the BLAST tool with the default parameters. The result showed that two neighboring upland cotton genes were hit: Gh_A07G1555 and *GhCAT7* (Gh_A07G1556), respectively (Figure S1). Additionally, the alignment result of the CDS of GbCAT7 (GOBAR_AA20422) and Gh_A07G1555 supported our speculation, indicating that the sequence of *GbCAT7* CDS annotated two genes in the upland cotton genome (Figure S2). Subsequently, we designed four primer pairs for PCR amplification of the actual *GbCAT7* ORF using the cDNA of the two-week-old Hai7124′s leaf as a template. The four forward primers (F, F1, F2 and F3, the primer sequence see Table S2) were located at the TSS of *GbCAT7* (GOBAR_AA20422), the upstream 31 bp of the first TSS that we conjectured, the first TSS that we conjectured, and the second TSS that we conjectured, respectively (Figure S1). Agarose gel electrophoresis showed that only the fourth primer pair (F2 and R) specifically amplified the objective band, suggesting that the actual TSS of *GbCAT7* was the ATG contained by the F2 forward primer. The PCR product was verified by sequencing (Figure S1). These results confirmed our speculation, and the assembly of GOBAR_AA20422 in the database (*G. barbadense* (AD2), NAU) contained two gene transcripts; one was *GbCAT7*, and the other was the orthologous gene of Gh_A07G1555.

### 3.3. Phylogenetic Analysis of CAT Gene Family

**Figure 2:**
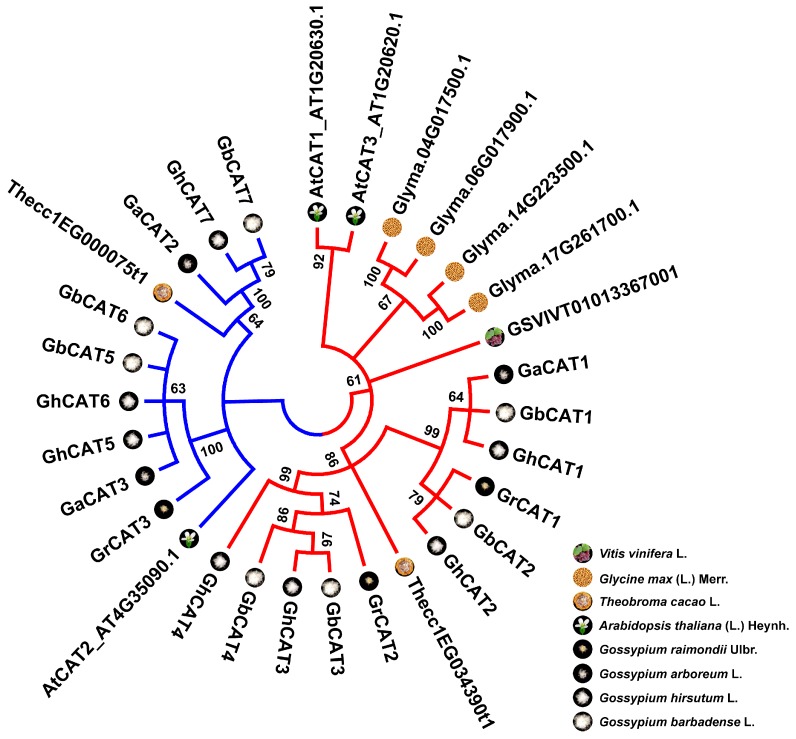
The neighbor-joining (NJ) phylogenetic tree of *CATs* in cotton, cacao, *Arabidopsis*, soybean, and grape. The tree was constructed with predicted full-length amino acid sequences of identified *CAT* genes from in *G. barbadense* L. (Gb), *G. hirsutum* L. (Gh), *G. arboreum* L. (Ga), *G. raimondii* Ulbr. (Gr), *A. thaliana* (Linn.) Heynh. (At), *T. cacao* L., *G. max* (Linn.) Merr. and *V. vinifera* L. Bootstrap values from 1000 replicates were shown above nodes. Branches corresponding to partitions reproduced in <60% of bootstrap replicates were collapsed in the phylogenetic trees. They were classified into two groups. Group I and II represented by red and blue, respectively.

To investigate the relationship of the plant evolution and *CAT* gene family expanding, using the same method identifying *CAT* genes in *Gossypium* genomes, we also searched for *CAT* genes in the genome of 4 other plants descended from the common eudicot genome ancestor in Rosids. Among these plants, the number of *CATs* was one in *Vitis vinifera* L., 4 in *G. max* (Linn.) Merr., 3 in *A. thaliana* (Linn.) Heynh., 2 in *T. cacao* L., 3 in *G. arboreum* L. and 3 in *G. raimondii* Ulbr., respectively (Figure S3 and Table S3). These *CAT* genes were clustered into two groups, which showed basic accordance with previous phylogenetic analyses of plant CATs (Figure 1A and Figure 2) [55]. Previous studies had revealed that, based on a phylogenetic analysis among prokaryotic and eukaryotic catalases, CATs could be classified into 3 clades. Clade 1 contained small subunit catalases from plants and a subset of bacteria; clade 2 contained large subunit catalases from fungi and a second subset of bacteria; and clade 3 contained small subunit catalases from bacteria, fungi, protists, animals, and plants [55].

Among the four species of *Gossypium*, the total *CAT* gene number of the two diploid cotton types, which were regarded as an A-genome ancestor and D-genome ancestor of the two allotetraploid cotton types, were basically equal to *G. hirsutum* L. and *G. barbadense* L. Additionally, the *CATs* consisting in the At-subgenome of upland cotton and sea-island cotton were mainly clustered with the *CATs* of *G. arboreum* L., and the *CATs* consisting in the Dt-subgenome were mainly clustered with the *CATs* of *G. raimondii* Ulbr. (Figure 2). The result was consistent with the hypothetical consequence of two diploid cotton species reunited geographically and followed by polyploidization to form the allopolyploid cotton types approximately 1–2 million years ago (Mya) [39,40], suggesting that the *CAT* gene family of allotetraploid cotton types might expand by the hybridization and subsequent polyploidization event.

In terms of the eight species in Rosids, the gene number and phylogeny of *CATs* were basically consistent with the genome paleo-history (Figure 2 and Figure S3). Rosids had been shown to evolve through rounds of paleo-polyploidy [56,57], and two types of events—an ancestral event (referenced as γ) and lineage-specific events (referenced as α, β and 1 to 4)—have been reported in the literature for Rosids (Figure S3). The result showed that the evolution and expanding of the *CAT* gene family in Rosids might be driven by whole-genome duplication (WGD), or polyploidy, followed by gene loss and diploidization.

### 3.4. Synteny Analysis of CAT Genes between Gossypium

**Figure 3:**
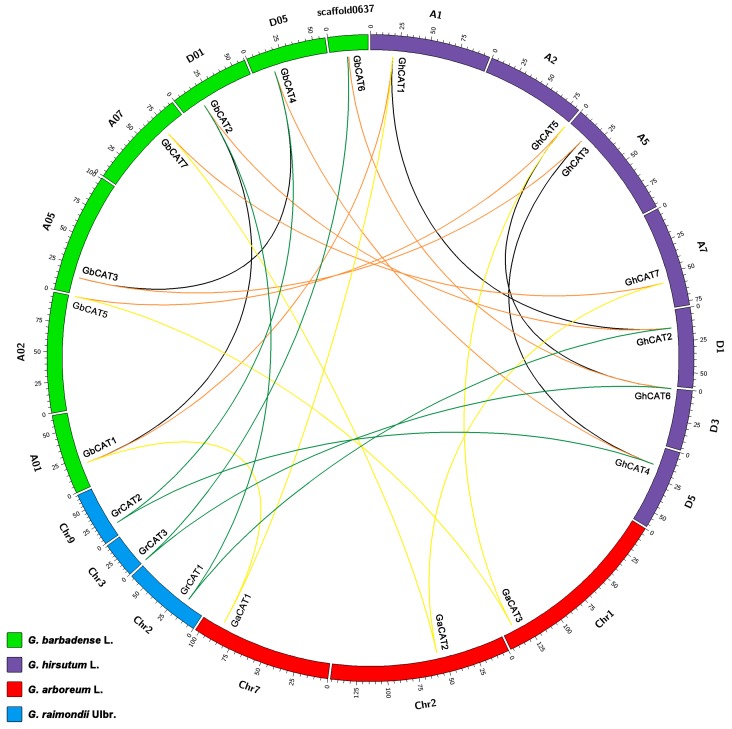
The synteny relationships of *CAT* genes among four sequenced cotton species. *G. arboreum* L., *G. raimondii* Ulbr., *G. hirsutum* L. and *G. barbadense* L. chromosomes are indicated in red, blue, purple, and green, respectively. The putative orthologous *CAT* genes between *G. arboreum* L. and two tetraploids species, between *G. raimondii* Ulbr. and two tetraploids species, and between *G. hirsutum* L. and *G. barbadense* L., are connected by yellow, dark green and orange lines, respectively. Black lines connect the putative paralogous genes.

To analyze the synteny and collinearity relationships of *CAT* genes between the two allotetraploid cotton types and two diploid cotton types, we identified the orthologous and paralogous genes among these released cotton genomes (Figure 3 and Table S4). Among the candidate upland cotton *CAT* genes, 6 *GhCATs* had orthologous genes in the two diploid cotton types, and 3 genes (*GhCAT1*/*5*/*7*) showed an A genome origin and 3 genes (*GhCAT2*/*4*/*6*) showed a D genome origin. Among the candidate sea-island cotton *CAT* genes, 6 *GbCATs* had orthologous genes in the two diploid cotton types, and 3 genes (*GbCAT1*/*5*/*7*) showed an A genome origin and 3 genes (*GbCAT2*/*4*/*6*) showed a D genome origin. Additionally, all 7 *GhCAT* genes were the orthologous genes of the 7 *GbCATs*, and there were no orthologous genes between the two diploid cotton types (Figure 3 and Table S4). In addition, to analyze the gene duplication events of the *Gossypium CAT* gene family, we characterized three paralogous gene pairs (*GhCAT1*/*2*, *GhCAT3*/*4* and *GhCAT5*/*6*) of *CATs* in the upland cotton genome, two pairs (*GbCAT1*/*2* and *GbCAT3*/*4*) in the sea-island cotton genome, and no paralogous genes in the two diploid cotton types (Figure 3). The duplicate genes of the *CAT* gene family in the two allotetraploid cotton types were all classified into WGD/segmental duplicates, and the other duplicate mechanisms which might make different contributions to evolution, such as tandem, proximal and/or dispersed duplications, were not detected (Figure 3 and Table S4). These results suggested that the WGD/segmental duplications might mainly contribute to the expansion of the *CAT* gene family during *Gossypium* evolution.

### 3.5. Expression Analysis of GhCATs and GbCATs in Different Tissues/Organs and Fiber Development Stages

**Figure 4:**
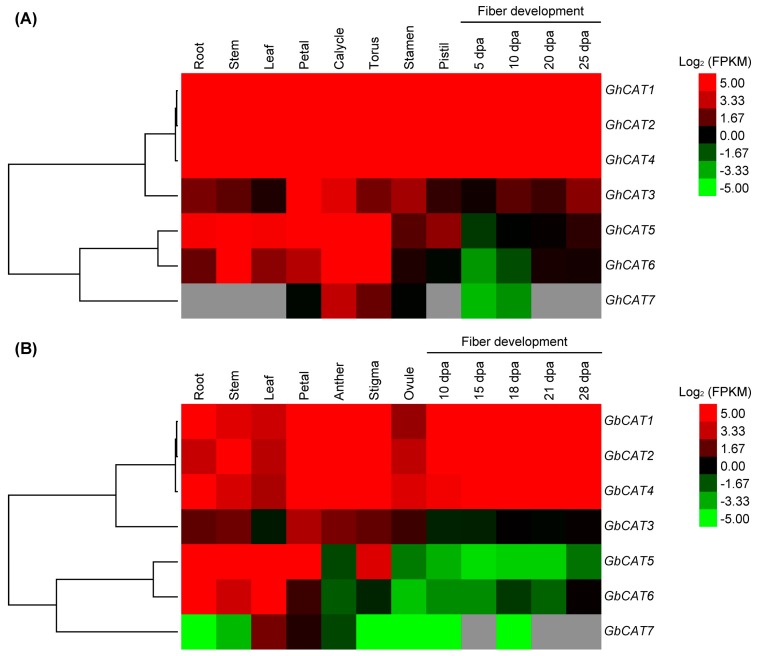
Transcriptional profiling of *GhCATs* and *GbCATs* in different tissues/organs and fiber development stages. The log_2_ of FPKMs (fragments per kilobase per million reads) values were calculated by RNA-Seq data to show the expression levels of the *CAT* genes in upland cotton and sea-island cotton. The colors indicated in the scale were represented by the relative expression levels. FPKMs data were obtained from the databases of ccNET (http://structuralbiology.cau.edu.cn/gossypium/) and CottonFGD (https://cottonfgd.org/). (**A**) The heat-map showed the hierarchical clustering of the relative expression of 7 *GhCATs* in root, stem, leaf, petal, calycle, torus, stamen, pistil, and fibers at 5, 10, 20 and 25 dpa. (**B**) The heat-map showed the hierarchical clustering of the relative expression of 7 *GbCATs* in root, stem, leaf, petal, anther, stigma, ovule, and fibers at 10, 15, 18, 21 and 28 dpa.

Gene functions closely correlated with gene expression patterns. To further investigate the biological functions of the *CAT* gene family in the two allotetraploid cotton types, we gathered the transcriptome data of different tissues/organs and fiber development stages that were published with the two-cotton whole genome sequencing [39,40] (Table S5). Generally, the expression levels of the upland cotton *CAT* gene family were higher than that of the sea-island cotton *CAT* genes; and among the members of the upland cotton *CAT* gene family, the genes clustering in group I (*GhCAT1*/*2*/*3*/*4*) showed higher expression levels than those in group II (*GhCAT5*/*6*/*7*), and sea-island cotton *CAT* genes had a similar expression pattern (Figure 4 and Table S5). Remarkably, *GhCAT3* and *GbCAT3*, which were in group I, showed a lower expression than other genes of group I; and, *GhCAT7* and *GbCAT7* were expressed at very low levels or even barely detectable in all the tissues/organs and fiber development stages (Figure 4 and Table S5). So, we suggested that *CAT1*/*2*/*4* of upland cotton and sea-island cotton can constitutively contribute catalase activity in normal conditions. Our findings indicated that the developmental and spatial-expression specificity of the *CAT* gene family might be involved in the growth and development of different tissues or organs in upland cotton and sea-island cotton.

### 3.6. Transcription Levels of GhCATs and GbCATs under V. dahliae Treatment

**Figure 5:**
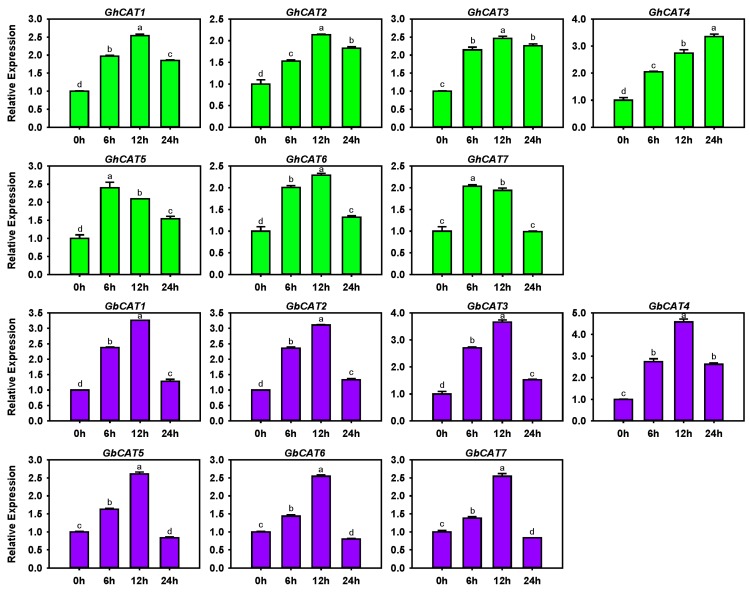
The expression patterns of *GhCATs* (filled green) and *GbCATs* (filled purple) under *V. dahliae* treatment. Different stress treatment times are shown on the *x*-axis and the relative expression levels on the *y*-axis. The expression levels of *CATs* are normalized to that of the cotton *UBQ7* gene and are compared with the control (0 h). The expression level was calculated using the 2^−ΔΔCT^ (ΔΔC_T_ = (C_T_, *CATs*—C_T_, *UBQ7*) treatments at different times—(C_T_, *CATs*—C_T_, *UBQ7*) 0 h) method. Error bars represented standard deviations of the mean values from three independent experiments. ANOVA (analysis of variance) was calculated using DPS and *p*  <  0.05 was considered statistically significant.

As previously reported, plant *CAT* genes played crucial roles in the resistance to pathogen infection [27]. To determine the functions of *CAT* genes in plant defenses against VW, we analyzed the expression patterns of the *CAT* gene family after *V. dahliae* strain Vd414 inoculation in upland cotton SF06 and sea-island cotton Hai7124, which exhibited susceptibility and resistance to *V. dahliae*, respectively (Figure 5). Further, these *CATs* were significantly induced by *V. dahliae* and quickly reached peak expression levels at different time points in the two allotetraploid cotton types (Figure 5). In addition, we found that the average C_t_ values of four *CAT* genes (*GhCAT3*, *GhCAT7*, *GbCAT3* and *GbCAT7*) were smaller than that of others during *V. dahliae* treatment (data not shown). This finding indicated that the four CAT genes showed a relatively low expression during *V. dahliae* treatment, which was basically consistent with the results showed in Figure S4 and Table S5. These data suggested that *CAT* genes might be involved in defense against *V. dahliae*, and the CAT-mediated regulating of ROS signaling might be related to the difference of resistance to VW between sea-island cotton and upland cotton. However, we need further investigations to verify which one or more genes among the family may be putative resistance components to VW and the regulatory mechanisms.

### 3.7. Predictions of Putative Molecular Regulatory Mechanisms of Cotton CATs

The previous parts have discussed the expression of cotton *CAT* gene family and their potential functions in VW resistance. However, how the steady-state levels of *CATs* are altered for mediating ROS metabolism is yet to be elucidated. In the present part, the predictions of transcriptional related components involving TFs and posttranscriptional regulation mediated by alternative splicing (AS) and miRNAs may provide an insight into the putative molecular regulatory mechanism of cotton *CATs* gene expression and their functional multiplicity.

#### 3.7.1. Prediction of Transcription Factor Binding Sites

**Figure 6:**
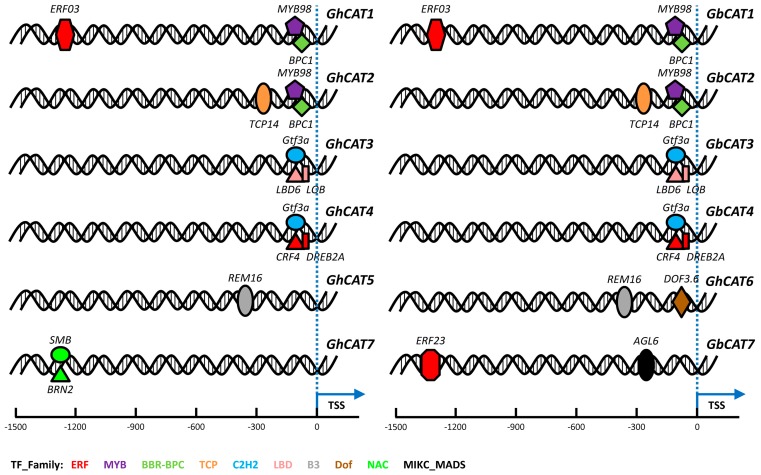
The schematic diagram of the highly conserved transcription factor binding sites (TFBSs) relative to that of the upland cotton and sea-island cotton *CAT* gene transcription start site (TSS). The information of potential TFBS was predicted using the Binding Site Prediction tool in PlantTFDB 4.0 with a stricter parameter: threshold *p*-value ≤ 1 × 10^−6^ (http://planttfdb.cbi.pku.edu.cn/).

TFs temporarily and spatially regulate the transcription of their target genes through binding certain upstream elements. To explore regulatory interactions between TFs and cotton *CATs*, we collected the upstream 1.5 kb genomic sequences relative to the TSS of upland cotton and sea-island cotton *CAT* genes as putative promoter regions and searched for highly conserved TFBSs in these promoter regions using the Binding Site Prediction server. In terms of upland cotton, 15 TFs belonging to 9 families might bind to the putative promoter regions of 7 *GhCATs* (*GhCAT1*/*2*/*3*/*4*/*5*/*6*/*7*). Like upland cotton *CATs*, the 13 TFs belonging to 7 families might bind to the 5 *GbCATs* (*GhCAT1*/*2*/*3*/*4*/*7*) (Figure 6 and Table S6). Among these TF families, many were also found in bacteria, yeast and animals, such as the C2H2, ERF, MYB and MADS families. However, other classes of transcription factors are plant-specific, such as the B3, BBR-BPC, Dof, LBD, NAC and TCP families. Numerous discoveries have emphasized that these TF families play crucial roles in plant stress response and during plant growth and development, such as in the carbohydrate metabolism, the regulation of the circadian clock, seed germination, gametophyte development, hormone pathways, leaf development, embryo development, flower development, cell cycle regulation, and response to biotic and abiotic stresses [58,59,60,61,62,63]. Furthermore, we carried out GO annotation of the TFs binding to the upland cotton and sea-island cotton *CAT* genes’ putative promoter regions based on their cellular location, molecular function, and biological process. The results showed that these TF families were involved in some metabolic process (GO:0009058, GO:0071704, GO:0044237, GO:0019222, GO:0006807 and GO:0044238) and in the regulation of the biological process (GO:0050789) and cellular process (GO:0009987) (Figure S5). The putative promoters of upland cotton and sea-island cotton *CAT* genes presented different types and numbers of TFBSs, which indicated that *CAT* genes might be regulated by these TF families at the transcriptional level and might be involved in some growth and development progresses as well as responding to various stresses, including VW pathogen infections.

#### 3.7.2. Survey of Alternative Splicing

**Figure 7:**
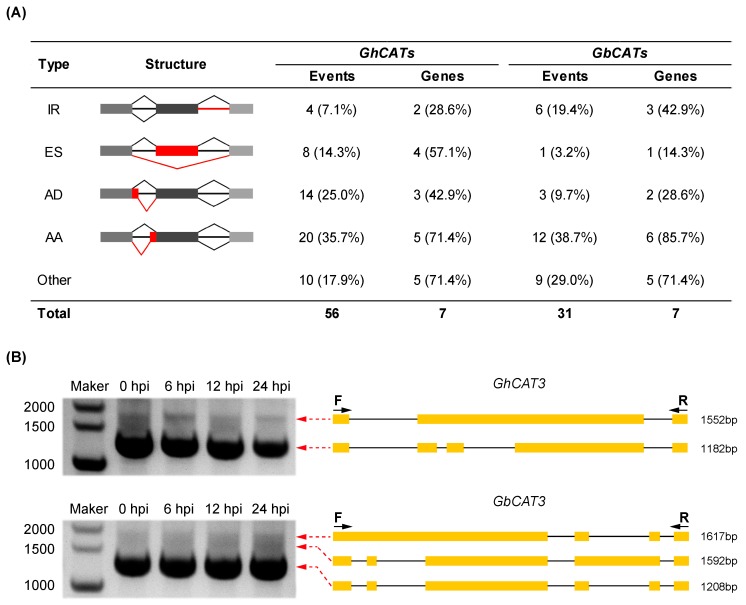
The characterization of alternative splicing (AS) events and the validation of full-length isoforms using the reverse transcription polymerase chain reaction (RT)-PCR. (**A**) Classification of AS events. Cartoons show AS events: intron retention (IR), exon skipping (ES), alternative 5′ donor sites (AD) and alternative 3′ acceptor sites (AA). The numbers of AS events and associated genes are shown. The numbers in the parentheses show the proportions of CATs occupying the gene family undergoing AS; (**B**) RT-PCR validation of AS events for *GhCAT3* and *GbCAT3*. Gel bands in each figure show the DNA makers and PCR results in four samples (hpi, hour post inoculation). The transcript structure of each isoform is shown in the right panel. Yellow boxes show exons and lines with arrows show introns. PCR prime pairs (F, forward and R, reverse) are shown on the isoforms of each gene. The length of each full-length isoform is shown after the transcript structure.

The AS of precursor mRNAs (pre-mRNAs), which generates multiple transcripts from a single gene, is an important modulator of gene expression that can increase transcriptome and proteome diversity [64]. It has been estimated that, in humans, ~95% of multi-exonic genes were alternatively spliced, and >60% of multiple exons containing genes in *Arabidopsis* were subject to AS [65]. In plants, this post-transcriptional mechanism is markedly induced in response to environmental stress, and recent studies have identified alternative splicing events that allow for rapid adjustment of the abundance and function of key stress-response components [66]. To explore the potential relationship between the AS of the *CAT* gene family and VW resistance in upland and sea-island cotton types, we analyzed potentially alternatively spliced isoforms by integration of the mRNA-seq data. Four major AS events were observed, including intron retention (IR), exon skipping (ES), alternative 5′ donor sites (AD) and alternative 3′ acceptor sites (AA). The statistics of the AS events of the *CAT* gene family and the corresponding gene models in *G. hirsutum* L. and *G. barbadense* L. were shown in Figure 7A and Figure S6. We found that all *CAT* genes underwent AS events and the number of AS events in *GhCATs* (56) was nearly twice as many as that in *GbCATs* (31) (Figure 7 and Figure S6). Compared with *CATs* clustering in group I, *CAT5* and *CAT6* had fewer potentially alternative spliced isoforms (Figure S6). Additionally, *CAT3* and *CAT7*, which expressed at very low levels or even barely detectable in the developmental stages and during stress treatments (Figure 4, Figure 5, and Figure S4), underwent more AS events. Hence, we inferred that AS might regulate the expression of the *CAT* gene family at the post-transcriptional level. In addition, splice junctions (SJs) were first characterized by investigating the terminal dinucleotide signature. We found that the GT-AG type occupied all of the intron borders and no other types, such as AT-AC and GC-AG (data not shown). Our findings indicated that the GT-AG type SJs might be associated with the production of different transcript isoforms.

We selected *GhCAT3* and *GbCAT3* to validate the accuracy of the AS events using RT-PCR with the corresponding designed primers (Table S2). We found that the size of each amplified fragment was consistent with that of the expected fragment (Figure 7B). The amplified fragments were then ligated into the *pEASY*^®^-Blunt (TransGen) for Sanger sequencing. We observed sequence consistency between the cloned fragments and predicted sequences based on the mRNA-seq data.

#### 3.7.3. Prediction of miRNA Target Sites

**Figure 8:**
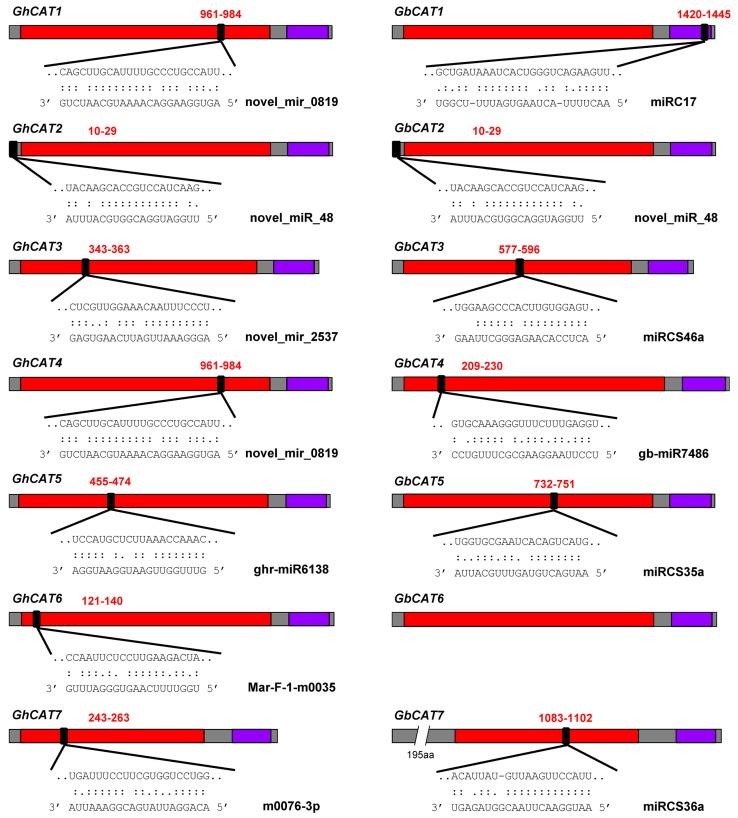
The miRNA-mediated targeting regulatory relationships of *CATs* in upland cotton and sea-island cotton. Heavy grey boxes represent the ORFs (open reading frames) of *CATs*. Catalase domain (*Catalase*, PF00199) and catalase-related immune-responsive domain (*Catalase-rel*, PF06628) are shown in red and purple boxes, respectively. miRNA complementary sites (black filling) with the nucleotide positions of *CATs* are indicated. The RNA sequence of each complementary site from 5′ to 3′ and the miRNA sequence from 3′ to 5′ are shown in the expanded regions.

To preliminarily explore the miRNA-mediated posttranscriptional regulatory mechanisms of the *CAT* gene family in upland cotton and sea-island cotton, we searched CDS for putative target sites of cotton miRNAs using the psRNATarget server. The results showed that 10 upland cotton miRNAs targeted 7 *GhCATs* and 8 sea-island cotton miRNAs targeted 6 *GbCATs* (Figure 8 and Table S7). As shown in Figure 8, *GhCAT1* and *GbCAT1* were targeted by novel_mir_0819 and miRC17 with sites in the *Catalase* domain and *Catalase-rel* domain, respectively; *GhCAT2* and *GbCAT2* were both targeted by novel_miR_48 with sites in the 5′-end of CDS; *GhCAT3* and *GbCAT3* were targeted by novel_mir_2537 and miRCS46a with sites both in the *Catalase* domain, respectively; *GhCAT4* and *GbCAT4* were targeted by novel_mir_0819 and miRCS46a/gb-miR7486 with sites both in the *Catalase* domain, respectively; *GhCAT5* and *GbCAT5* were targeted by ghr-miR6138 and miRCS35a with sites both in the *Catalase* domain, respectively; *GhCAT6* was targeted by Mar-F-1-m0035 with the site in the *Catalase* domain; *GhCAT7* and *GbCAT7* were targeted by m0076-3p and miRCS36a with sites both in the *Catalase* domain, respectively (Figure 8). In addition to the target sites mentioned above, we also found that upland cotton novel_mir_0819 targeted the *GhCATs* in group I (*GhCAT1*/*2*/*3*/*4*) with the site in the *Catalase* domain (Figure 1). Upland cotton ghr-miR6138 and Mar-F-1-m0035 both targeted *GhCAT5* and *GhCAT6* in the *Catalase* domain. Additionally, novel_miR_48 that was both probably in upland cotton and sea-island cotton and induced by *V. dahliae* inoculation [48], targeted *GhCAT2* and *GbCAT2* (Table S7). Our findings suggested that the miRNA-mediated regulation of the *CAT* gene family was possibly conserved in the two allotetraploid cotton types, but the actual posttranscriptional regulatory relationships between cotton miRNAs and *CATs* need to be detected and verified in further experiments through relative molecular biology techniques.

#### 3.7.4. Regulation of Isoforms by miRNAs

To investigate how miRNAs interact with isoforms, we predicted the target sites for the two allotetraploid cotton miRNAs using all *CAT* full-length isoforms. In upland cotton, 47 isoforms from all 7 *GhCAT* genes (including a total of 48 isoforms) were identified to be putative targets of 14 ghr-miRNAs, and only one isoform transcribed from *GhCAT2* was not targeted by the miRNAs (Table S8). In sea-island cotton, we also found that 27 isoforms from 6 *GbCAT* genes (including a total of 30 isoforms) were putative targets of 11 gb-miRNAs, and only one isoform transcribed from *GbCAT2* was not targeted by the miRNAs (Table S8). In addition, *GbCAT6* was not transcript targeted by miRNAs. Our findings indicated that some isoforms transcribed from the same gene might escape regulation by miRNAs.

We also examined the effect of AS on the gain/loss of miRNA target sites among all CAT isoforms regulated by miRNAs, and all examples associated with miRNAs were shown in Figure S6. Specifically, *GhCAT1* was predicted with nine isoforms, of which five and eight were targets of novel_miR_48 and novel_mir_0819, respectively (Figure S6). This gene underwent an ES event to produce three and one isoforms losing the novel_miR_48 and novel_mir_0819 target site, respectively. *GhCAT2* transcribes nine isoforms, of which four were found to lose the target sites of novel_miR_48, ghr-miR5298, ghr-miR163, novel_mir_0819 and ghr-miR7502bp from an ES and/or AD event. *GhCAT3* was found to transcribe four isoforms, of which one gained the target site of ghr-miR2645 by an IR event. *GhCAT4* was found to transcribe eight isoforms, of which two gained the target sites of m0028 by an AA event. *GhCAT7* was predicted to have fourteen isoforms, of which one and five gained the m0356 and m0149 target sites by an IR event and an AS event, respectively (Figure S6). *GbCAT2* transcribes three isoforms, of which one was found to lose the target sites of novel_miR_48 by an ES event. *GbCAT3* was found to transcribe seven isoforms, of which two gained the target sites of gb-miR7486 by an IR event. *GbCAT7* was predicted to have five isoforms, of which two and two gained the miRC14 and miRC25 target sites by an AA event and anAS event, respectively (Figure S6). Collectively, these results indicated that miRNAs can couple with AS to regulate gene expression at the isoform level.

## 4. Discussion

### 4.1. Evolutionary History of the CAT Gene Family in Gossypium Spp.

**Figure 9:**
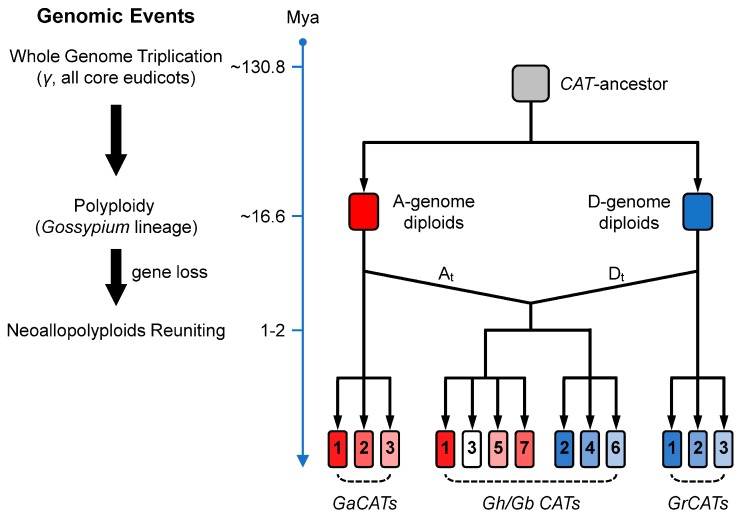
The scheme of the *CAT* gene family loci during the evolutionary history of *Gossypium* spp. The hypothetic succession of genomic events occurring at or including cotton *CATs* was proposed on the right. The evolution time was given from the top to the bottom in Mya. The scheme of the emergence of the 7 cotton *CATs* genes was given at the left.

In the present study, we identified 7 *GhCAT* genes and 7 *GbCAT* genes from the genomes of upland cotton and sea-island cotton, respectively. We also searched *CAT* genes in two sequenced diploid cotton types and four other Rosids plant genomes, which were at key evolutionary nodes. The results showed that the gene number and phylogeny of *CATs* were basically consistent with the genome paleo-history (Figure S3), which suggested that these plant genomes have become more complexed during evolution and the *CAT* gene family expanded. Then, we identified genomic events in the progress of Rosids plants and investigated the synteny and collinearity relationships of *CAT* genes in *Gossypium* spp. The results indicated that WGD or polyploidy events had played a major role in the *CAT* gene family expansion in the Rosids. By combining several lines of evidence provided by phylogenetic analysis, and gene synteny for large-scale identification of *CAT* genes within eight Rosids genomes (Figure 3 and Figure S3), we built a tentative evolution model of the *CAT* gene family loci during the evolutionary history of *Gossypium* spp. (Figure 9). In the model, *Gossypium CATs* descended from one common ancestor, which expanded in diploid cotton types by one penta- or hexa-ploid duplication event in the *Gossypium* lineage after splitting from the cacao lineage ~16.6 Mya. Then, through the evolution of the cotton species following the gene loss, the *GaCAT* and *GrCAT* gene families formed, containing 3 members respectively. On the other side, when two diploid cotton species reunited geographically and were followed by polyploidization to form the allopolyploid cotton approximately 1–2 Mya, the *CAT* gene families of A-genome diploids and D-genome diploids also combined and formed the expanding gene family in neoallopolyploids. Among the *GhCATs* and *GbCATs*, *CAT1*/*5*/*7*, located on the At-subgenome, originated from *GaCAT1*/*3*/*2*, respectively; *CAT2*/*4*/*6*, located on the Dt-subgenome, originated from *GrCAT1*/*2*/*3*, respectively. Additionally, we propose that the donor gene of *GhCAT3*/*GbCAT3* was lost during the evolution of the D-genome diploids (Figure 9).

### 4.2. Cotton CAT Genes Probably Function in Defense against Biotic Stress by Mediating ROS Metabolism

CAT is a strong antioxidant enzyme that mediates the ROS-scavenging process and thus plays a critical role in the development and plant responses to abiotic stresses [67]. It has been investigated in many plant species, including *Arabidopsis* [68,69,70,71], rice [9,14,72], cucumber [16,73], sugarcane [74] and sweet potato [75]. In *Arabidopsis*, drought-, and salt stress-induced *AtCAT1* expression was mediated by an *Arabidopsis* MAPK kinase AtMEK1 and, furthermore, the triggering of ROS production might be involved in these processes [69]. Transcription of *AtCAT2* was repressed by WRKY75 to enhance the ROS accumulation to accelerate leaf senescence [68]. Expression of *AtCAT2* was suppressed to trigger endogenous ROS production in response to Pb stress [70]. *AtCAT3* was phosphorylated by a calcium-dependent protein kinases CPK8 to mediate ROS homeostasis in the peroxisome of guard cells and was associated with a classical drought stress response, and the overexpression of *AtCAT3* enhanced its tolerance to drought stress [71]. In rice, *OsCatA*, *OsCatB*, and *OsCatC* were involved in the environmental stress response; the regulation of ROS levels or homeostasis related to root growth regulation and photorespiration; and the overexpression of *OsCatA* and *OsCatC* conferred tolerance to drought stress in transgenic rice [14]. In the stress tolerant genotype of rice, *OsCatA* and *OsCatB* were the most responsive to high salinities and cold, while in the sensitive genotype, *OsCatA* and *OsCatC* responded positively to saline stress, as did *OsCatA* and *OsCatB* to low temperatures [72]. *OsCatC* was phosphorylated and activated by a salt tolerance receptor-like cytoplasmic kinase to regulate ROS homeostasis and improve salt tolerance in rice [9]. In cucumber, *CsCAT1* was only activated by abscisic acid treatment, *CsCAT2* transcription was reduced in response to droughts, *CsCAT3* transcription was enhanced under salt and drought stress conditions and reduced under ABA stress conditions, and the overexpression of *CsCAT3* in *Escherichia coli* could increase the tolerance to heat, cold, salinity and osmotic conditions. These results indicated that the activation of cucumber *CsCATs* might differ in response to different abiotic stresses [16,73]. In sugarcane, the expression of *ScCAT1* can be induced by different kinds of stresses, including oxidative, heavy metal, drought and NaCl stresses [74]. In sweet potato, the expression of *IbCAT2* was regulated by PEG6000 and NaCl treatments, and the overexpression of *IbCAT2* conferred salt and drought tolerance in *E. coli* and *Saccharomyces cerevisiae* [75]. Taken together, these studies suggest that CATs were important in the triggering of the ROS metabolism, and the activation of plant responses to abiotic stresses. However, little research was carried out to investigate the relationship between CAT and the *V. dahliae* infection in cotton.

In the present study, to investigate the roles of the cotton *CAT* gene family in responses to biotic stress, we analyzed their transcript levels under the *V. dahliae* infection treatment and found that the expression of the cotton *CAT* gene family was up-regulated, which might be involved in the defense against *V. dahliae* (Figure 5). As for which member(s) mainly contributed to the biotic stress response preliminarily, the functional analysis of cotton *CAT* gene family needs to be further explored using the assays of over-expression and loss of functions.

### 4.3. The Potential Regulatory Mechanisms of Cotton CAT Gene Expression

Based on the results presented here as well as those reported previously [10,11,24], CAT-dependent ROS metabolism plays important functional roles in response to various stresses, including abiotic and biotic stresses. There were some publications that described the regulation of *CATs* [76]. However, the potential regulatory mechanisms of *CAT* gene expression are yet to be elucidated in cotton. In the resent study, we predicted the TFBSs, AS events and miRNA-mediated regulations which might provide an insight into the potential regulatory mechanisms of cotton *CAT* gene expression and their functional multiplicity.

Through regulating target gene expression accurately, TFs, which generally consisted of a DNA-binding domain and transcriptional activation (or inhibition) domain, operate as important switches of transcription networks and control multiple cellular processes [77], including plant growth and development [58,62,68], and response to stresses [78]. We predicted the TFBSs in the promoter regions of the cotton *CAT* gene family and found that some famous plant TFs might bind to cotton *CAT* genes and potentially regulate their expression. These TFs belonged to different families such as MYB, NAC and TCP (Table S6, Figure 6 and Figure S5). In *Arabidopsis*, Guo et al. found that WRKY75, a WRKY TF, repressed ROS scavenging by suppressing *CAT2* transcription involved in the regulation of leaf senescence [68]. In rice, a recent study provided the rare combination of TFs and ROS burst that constituted a novel molecular mechanism employed by rice blast resistance [78]. More concretely, a natural allele of a C_2_H_2_-type transcription factor causes a single nucleotide mutation in the promoter, which resulted in the reduced expression of the gene through the binding of the repressive MYB transcription factor and, consequently, an inhibition of ROS degradation by decreasing the peroxide accumulation and enhanced disease resistance [78]. In cotton, recent studies have demonstrated that by regulating ROS metabolism networks, many of these TFs were involved in different aspects of plant developmental processes, including leaf senescence [79], anther/pollen development [80] and fiber development [81], as well as in stress responses, such as drought tolerance [82], salt tolerance [83] and pathogen inoculation [84]. However, how CAT-mediated ROS metabolism is regulated by TFs involved in both abiotic and biotic stresses in cotton remains unknown and needs to be further investigated in our following research with the help of newly developed technologies, such as GWAS, ATAC-seq, FAIRE-seq and ChIP-seq. It is a topic that we plan to report on comprehensively elsewhere.

In the present study, we found that under non-stress and stress conditions, the expression levels of cotton *CAT3* and *CAT7* were extremely low. Phylogenetic and synteny analysis revealed that the evolution of *CAT3* and *CAT7*, clustering to group I and group II, respectively, might be driven by gene duplication. It was generally known that gene redundancy, which most often resulted from gene duplication, generally had overlapping functions. Thus, we suspected that *CAT3* and *CAT7* were disrupted by a mechanism and there could only be a little effect on phenotype because of gene redundancy. Meanwhile, we noticed that compared with other *CATs*, *GhCAT7*, *GbCAT3* and *GbCAT7* existed more putative in AS events (Figure S6). Hence, we hypothesized that AS might be one of the potential mechanisms in the regulation of *CAT3* and *CAT7* expressions. AS of pre-mRNAs from multiexon genes allows for organisms to increase their coding potential and regulate their gene expression through multiple mechanisms [85]. Compared with constitutive splicing, more alternatively spliced isoforms exhibited cell-, tissue- or condition-specific expression patterns, and AS often generated transcripts with premature termination codons (PTC), which would be selectively degraded by a non-sense-mediated mRNA decay (NMD) process [86]. NMD was a cytoplasmic RNA degradation system, which occurs on ribosomes on the first round of translation. NMD triggered transcript decay when translation termination was perturbed [85]. During cloning ORFs of *CATs*, apart from *CAT3* and *CAT7*, the other *CAT* genes were cloned correctly from cDNA libraries of cotton infected by *V. dahliae*. Additionally, we had obtained multiple types of transcripts of *CAT3* and *CAT7*, which were all with PTC. Take the upland cotton example, 24 and 48 clones of *GhCAT3* and *GhCAT7* were randomly selected to be sequenced, respectively, and 2 and 14 types transcripts were obtained. Sanger sequencing showed that all these alternatively spliced isoforms contained PTC. We selected the largest-rate transcripts of *GhCAT3* and *GhCAT7* shown in Figure S8. According to our findings, the coupling of AS to mRNA stability through NMD might be one of the reasons why the expression levels of cotton *CAT3* and *CAT7* were extremely low under normal conditions and under stress treatments.

miRNAs, a class of single-stranded noncoding small RNAs, have been shown to be regulatory of gene expression by targeting endogenous and/or exogenous genes [87,88]. Growing evidence reveals that miRNA-guided regulation of ROS-related genes at the post-transcriptional level is essential for normal growth and development [89] and the adaptation to stress conditions [48,90] including drought, salinity, high light, heavy metals, and many bacterial and fungal pathogen infections. However, seldom reports had been found on the *CAT* gene expression and regulation mediated by miRNAs under *V. dahliae* infection. In our study, we predicted the miRNA-mediated posttranscriptional regulation of cotton *CATs* and found some putative target sites of cotton miRNAs (Table S7 and Figure 8). To preliminarily verify the target relationship of cotton *CATs* and miRNAs, we tested their expression patterns with Vd414 infection treatment by qPCR (Figure S7). There were 4 ghr-miRNAs and 2 gb-miRNAs and its target cotton *CAT* mRNAs showed a negative regulation relationship during the *V. dahliae* infection, respectively. This indicated that these cotton miRNAs might play crucial roles in the VW pathogen infection by regulating the *CAT* genes.

Recently, attention has been focused on mobile miRNAs that mediate cross-kingdom regulation in plant-pathogen interactions [87,88]. miRNAs from the plant pathogenic fungus, such as *Botrytis cinerea* Pers. and *Puccinia striiformis* f. sp. *tritici* Westend., targeted host mRNAs to suppress plant immunity during infection [91,92], and host-induced gene silencing (HIGS) that targeted dicer-like mRNAs in *B. cinerea* reduced the pathogen virulence [93]. Conversely, plant miRNAs were exported from cotton into the fungal pathogen *V. dahliae* to inhibit pathogen virulence gene expression to enhance the disease resistance of cotton plants [94]. Although miRNA-mediated bidirectional cross-kingdom regulation existed widely in plant-pathogen interactions, a genome-scale characterization analysis and the relationship between cross-kingdom regulation and ROS metabolism have not yet been reported. In view of this, combining previous studies of our laboratory [45,48], we constructed the sRNA library of *V. dahliae* cultured from hyphae recovered from infected cotton plants to investigate the topic using a high-throughput sequencing technology and the bioinformatics analysis. Some preliminary results have been obtained, and it is a topic that we plan to report on comprehensively elsewhere.

Recently, with the development of omics, high-throughput sequencing and bioinformatics, the arsenal of modern molecular biology technology was significantly expanded with iTRAQ, Hi-C, GWAS, CRISPR-mediated gene editing, and so on and so forth. These weapons had been applied to many areas of agriculture and life sciences, and important progress has been made. In fact, plant growth and development, disease resistance and the interactions between plants and their environment were regulated by many molecular mechanisms and metabolic pathways, which formed a complex and interrelated network. Therefore, we thought that finding out how to integrate these advanced technologies organically to investigate complex networks would reveal the nature of life in larger scales, though there is still a long way to go.

## 5. Conclusions

Various studies suggested that ROS, as the second cellular messengers of plants, played a crucial role in a variety of biotic stress responses. CAT, the first antioxidant enzyme to be discovered and characterized, maintained the balance of cellular redox biology by scavenging ROS. Here, we focused on the cotton *CAT* gene family, which coded CAT proteins and was involved in ROS metabolism by catalyzing H_2_O_2_ to water and oxygen. We identified 7 and 7 *CAT* genes in upland cotton and sea-island cotton genomes, respectively. WGD or polyploidy events contributed to the expansion of the *Gossypium CAT* gene family during the evolutionary process. *Gossypium CAT* genes showed different expression patterns in different tissues and developmental stages and under different stress treatments. Moreover, we predicted the putative molecular regulatory mechanisms of the *Gossypium CAT* gene family. Based on the analysis and preliminary verification results, we hypothesized that the *CAT* gene family might be regulated by TFs, AS events and miRNAs at different levels. Generally, our analysis of the *Gossypium CAT* gene family broadens our insight into the roles of *CAT* genes in plant stress responses and the ROS metabolism and provides the foundation for further functional characterization of the *GhCAT* and *GbCAT* gene families and for potential applications towards the genetic improvement of cotton.

## Figures and Tables

**Table 1:** The details of two allotetraploid cotton *CAT* genes.

Gene Name	Locus ID	Gene Features		Transcript Features			Protein Statistics				
		Genomic Position	Length (bp)	CDS Length (bp)	CDS GC Content (%)	Exon Number	Protein Length (aa)	Molecular Weight (kDa)	Isoelectric Point (*pI*)	GRAVY	Predicted Subcellular Localization
*GhCAT1*	Gh_A01G0845	A01:19440294-19443475 −	3182	1479	46.9	8	492	56.829	7.173	−0.533	Peroxisome
*GhCAT2*	Gh_D01G0873	D01:14348913-14352092 −	3180	1479	47.0	8	492	56.817	7.173	−0.538	Peroxisome
*GhCAT3*	Gh_A05G1539	A05:15642154-15645557 −	3404	1419	46.1	9	472	54.706	7.068	−0.561	Peroxisome
*GhCAT4*	Gh_D05G1710	D05:15409889-15413267 −	3379	1479	46.5	8	492	56.957	7.413	−0.587	Peroxisome
*GhCAT5*	Gh_A02G1698	A02:83178810-83181326 +	2517	1470	45.4	7	489	56.528	7.454	−0.546	Peroxisome
*GhCAT6*	Gh_D03G0021	D03:171438-173963 −	2526	1485	45.5	7	494	57.285	7.310	−0.543	Peroxisome
*GhCAT7*	Gh_A07G1556	A07:57094046-57096383 +	2338	1230	46.0	9	409	46.962	6.869	−0.498	Peroxisome
*GbCAT1*	GOBAR_AA22711	A01:19685673-19688948 −	3276	1479	47.0	8	492	56.845	7.173	−0.539	Peroxisome
*GbCAT2*	GOBAR_DD08701	D01:16738967-16742318 +	3352	1479	47.0	8	492	56.817	7.173	−0.538	Peroxisome
*GbCAT3*	GOBAR_AA35088	A05:14744651-14749782 −	5132	1380	46.7	9	459	53.290	7.251	−0.651	Peroxisome
*GbCAT4*	GOBAR_DD30012	D05:16498614-16501993 +	3380	1479	46.6	8	492	56.924	7.294	−0.585	Peroxisome
*GbCAT5*	GOBAR_AA31934	A02:98649356-98652362 +	3007	1479	45.4	8	492	56.903	7.453	−0.557	Peroxisome
*GbCAT6*	GOBAR_AA38517	scaffold_0637.UKA:19604-22605 +	3002	1479	45.6	8	492	56.939	7.453	−0.567	Peroxisome
*GbCAT7*	GOBAR_AA20422	A07:71313165-71323809 +	10645	1806	45.5	9	601	67.504	6.286	−0.662	Peroxisome

Gb: *G. barbadense* L.; Gh: *G. hirsutum* L.; CAT: catalase; GRAVY: grand average of hydropathy.

## Supplementary Materials

The following are available online at http://www.mdpi.com/2073-4409/8/2/86/s1, Table S1: Summary of some plant *CAT* genes in relation to the biotic and abiotic stress resistance, Table S2: Primers used in this study, Table S3: Gene numbers of *CAT* gene family in four other genomes descended from common eudicot genome ancestor with *Gossypium* in Rosids, Table S4: Orthologous *CAT* gene pairs among *G. arboreum* L., *G. raimondii* Ulbr., *G. hirsutum* L. and *G. barbadense* L., Table S5: The details of *GhCAT* and *GbCAT* gene family expression difference in different tissues and/or organs, at developmental processes of ovules and fibers, and under different stress treatments, Table S6: List of identified TFBSs in the putative promoter regions of *GhCAT* and *GbCAT* gene family using PlantTFDB 4.0 (http://planttfdb.cbi.pku.edu.cn/), Table S7: The predicted details of miRNA-mediated targeting regulatory relations with the principle transcripts of *CAT* gene family in upland cotton and sea-island cotton using the psRNATarget web server (http://plantgrn.noble.org/psRNATarget/home), Table S8: The predicted details of miRNA-mediated targeting regulatory relations with all full-length isoforms of *CAT* gene family in upland cotton and sea-island cotton using the psRNATarget web server (http://plantgrn.noble.org/psRNATarget/home). Figure S1: (A) Blast hits of *GbCAT7* (GOBAR_AA20422) in the upland cotton genome database [cDNA, *G. hirsutum* (AD1), NAU] and the location of the designed primer pairs for cloning the actual ORF of *GbCAT7*. (B) PCR result shows that the actual TSS of *GbCAT7* is the ATG contained by the F3 forward primer (green dotted underline in A). (C) Verification of the amplified line in Figure S1 (B) by PCR-based sequencing, Figure S2: The supporting alignment result of the coding sequence of *GbCAT7* (GOBAR_AA20422) and Gh_A07G1555. Query, the coding sequence of *GbCAT7*; Subject, the coding sequence of Gh_A07G1555, Figure S3: Phylogenetic tree of *Gossypium* and four other genomes descended from common eudicot genome ancestor in Rosids and the number of *CAT* genes identified from the genomes of these plants, Figure S4: Transcriptional profiling of *GhCATs* under different abiotic stress treatments, Figure S5: Gene Ontology (GO) annotation of the TFs binding to the upland cotton and sea-island cotton *CAT* genes’ putative promoter regions based on their cellular component, molecular function, and biological process, Figure S6: All potentially alternative spliced isoform structure of *GhCATs* and *GbCATs* full-length transcripts and identification of full-length isoforms targeted by cotton miRNAs, Figure S7: The relative expression levels of miRNAs and their putative targeting *CAT* genes under *V. dahliae* infection treatment, Figure S8: (A, B) Amplification of *GhCAT3* (Gh_A05G1539) and *GhCAT7* (Gh_A07G1556) by PCR with the designed primer pairs for cloning their transcripts. PCR results were aligned with gene structure annotation and were verified by Sanger sequencing.
