# Supplementary material for: The Catalase Gene Family in Cotton: Genome-Wide Characterization and Bioinformatics Analysis
Source: Cells. 2019 Jan 24;8(2):86. doi: 10.3390/cells8020086 (PMC6406514; doi:10.3390/cells8020086)
Supplement: Supplementary file 1 [file cells-08-00086-s001.zip › Supplementary Files Figure S1-S8.pdf]

Supplementary Files

The Catalase Gene Family in Cotton: Genome-Wide Characterization and Bioinformatics Analysis

Wei Wang, Yingying Cheng, Dongdong Chen, Dan Liu, Mengjiao Hu, Jie Dong, Xiaopei Zhang, Lirong Song and Fafu Shen *

Contents

Supplementary file 1: Table S1. Summary of some plant *CAT* genes in relation to the biotic and abiotic stress resistance.

Supplementary file 2: Table S2. Primers used in this study.

Supplementary file 3: Table S3. Gene numbers of *CAT* gene family in four other genomes descended from common eudicot genome ancestor with *Gossypium* in Rosids.

Supplementary file 4: Table S4. Orthologous *CAT* gene pairs among *G. arboreum* L., *G. raimondii* Ulbr., *G. hirsutum* L. and *G. barbadense* L..

Supplementary file 5: Table S5. The details of *GhCAT* and *GbCAT* gene family expression difference in different tissues and/or organs, at developmental processes of ovules and fibers, and under different stress treatments.

Supplementary file 6: Table S6. List of identified TFBSs in the putative promoter regions of *GhCAT* and *GbCAT* gene family using PlantTFDB 4.0 (http://planttfdb.cbi.pku.edu.cn/).

Supplementary file 7: Table S7. The predicted details of miRNA-mediated targeting regulatory relations with the principle transcripts of *CAT* gene family in upland cotton and sea-island cotton using the psRNATarget web server (http://plantgrn.noble.org/psRNATarget/home).

Supplementary file 8: Table S8. The predicted details of miRNA-mediated targeting regulatory relations with all full-length isoforms of *CAT* gene family in upland cotton and sea-island cotton using the psRNATarget web server (http://plantgrn.noble.org/psRNATarget/home).

(***All the tables in additional file see a separate excel file.***)

Supplementary file 9: Figure S1. **(A)** Blast hits of *GbCAT7* (GOBAR_AA20422) in the upland cotton genome database [cDNA, *G. hirsutum* (AD1), NAU] and the location of the designed primer pairs for cloning the actual ORF of *GbCAT7*. **(B)** PCR result shows that the actual TSS of *GbCAT7* is the ATG contained by the F3 forward primer (green dotted underline in A). **(C)** Verification of the amplified line in Figure S1 (B) by PCR-based sequencing.

Supplementary file 10: Figure S2. The supporting alignment result of the coding sequence of *GbCAT7*

(GOBAR_AA20422) and Gh_A07G1555. Query, the coding sequence of *GbCAT7*; Subject, the coding sequence of Gh_A07G1555.

Supplementary file 11: Figure S3. Phylogenetic tree of *Gossypium* and four other genomes descended from common eudicot genome ancestor in Rosids and the number of *CAT* genes identified from the genomes of these plants.

Supplementary file 12: Figure S4. Transcriptional profiling of *GhCATs* under different abiotic stress treatments.

Supplementary file 13: Figure S5. Gene Ontology (GO) annotation of the TFs binding to the upland cotton and sea-island cotton *CAT* genes' putative promoter regions based on their cellular component, molecular function, and biological process.

Supplementary file 14: Figure S6. All potentially alternative spliced isoform structure of *GhCATs* and *GbCATs* full-length transcripts and identification of full-length isoforms targeted by cotton miRNAs.

Supplementary file 15: Figure S7. The relative expression levels of miRNAs and their putative targeting *CAT* genes under *V. dahliae* infection treatment.

Supplementary file 16: Figure S8. **(A**, **B)** Amplification of *GhCAT3* (Gh_A05G1539) and *GhCAT7* (Gh_A07G1556) by PCR with the designed primer pairs for cloning their transcripts. PCR results were aligned with gene structure annotation and were verified by Sanger sequencing.

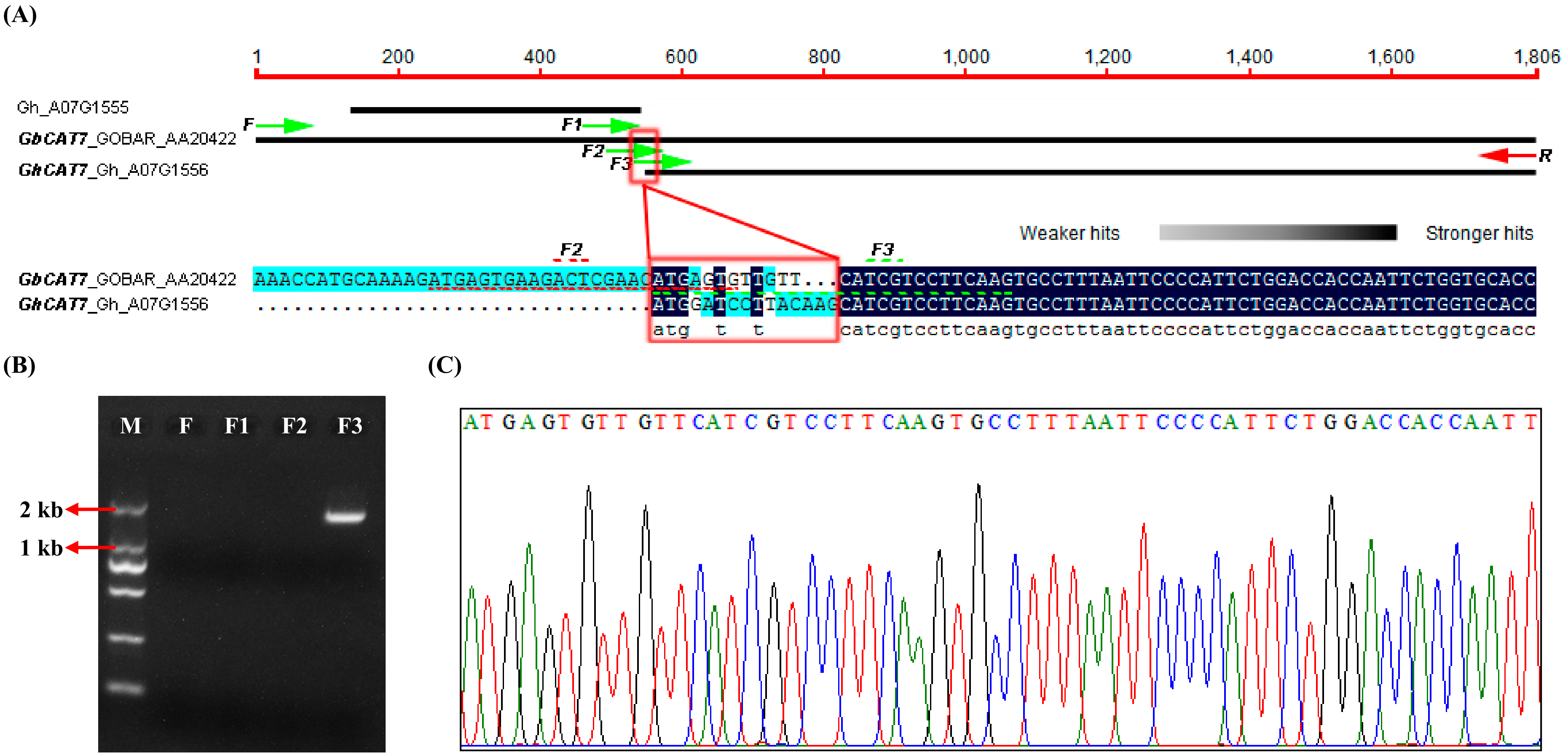

Supplementary file 9: Figure S1. **(A)** Blast hits of *GbCAT7* (GOBAR_AA20422) in the upland cotton genome database [cDNA, *G. hirsutum* (AD1), NAU] and the location of the designed primer pairs for cloning the actual ORF of *GbCAT7*. **(B)** PCR result shows that the actual TSS of *GbCAT7* is the ATG contained by the F3 forward primer (green dotted underline in A). **(C)** Verification of the amplified line in Figure S1 (B) by PCR-based sequencing.

Gh_A07G1555.1 LOC:A07|57085879..57086511|+;

1. Score	E value	Identities	Gaps	Strand
742.47 (822)	$0.00 \times 10^{+00}$	411/411 (100.00)	0/411 (0.00)	+/+

```
Query   139  ATGAGCATTGATGACGCTAATGAGACCGGGATAGATGATCCGAAGGGCCAGGGTTCTTCC  198
             ||||||||||||||||||||||||||||||||||||||||||||||||||||||||||||
Subject   1  ATGAGCATTGATGACGCTAATGAGACCGGGATAGATGATCCGAAGGGCCAGGGTTCTTCC  60
Query   199  TCTAGAAAAAGAGGGAGCACTATTGATGTTGCTATTCAGATTGGGTCTGGCAGTGGCAGT  258
             ||||||||||||||||||||||||||||||||||||||||||||||||||||||||||||
Subject  61  TCTAGAAAAAGAGGGAGCACTATTGATGTTGCTATTCAGATTGGGTCTGGCAGTGGCAGT  120
Query   259  GGATCAAATGCAAGGAAGAAAAGGGAAATGGAAATTCCAAGAGGTGAACCTACATGTTAC  318
             ||||||||||||||||||||||||||||||||||||||||||||||||||||||||||||
Subject 121  GGATCAAATGCAAGGAAGAAAAGGGAAATGGAAATTCCAAGAGGTGAACCTACATGTTAC  180
Query   319  GTATGTAGTAAAAATTTCACGTCCTGGAAGGCGGTGTTCGGGCATCTGAAATCCCATCAA  378
             ||||||||||||||||||||||||||||||||||||||||||||||||||||||||||||
Subject 181  GTATGTAGTAAAAATTTCACGTCCTGGAAGGCGGTGTTCGGGCATCTGAAATCCCATCAA  240
Query   379  AGGGAAACCCCAGGGGCATTGCCTCCGCCAACATTTACGCCAACTGAGGGCTCTCCTGAA  438
             ||||||||||||||||||||||||||||||||||||||||||||||||||||||||||||
Subject 241  AGGGAAACCCCAGGGGCATTGCCTCCGCCAACATTTACGCCAACTGAGGGCTCTCCTGAA  300
Query   439  AATAATAATGATGATGAAACTAACCCGAGGGAGCAACTAGCTCCCACTCTGTTGAACTTG  498
             ||||||||||||||||||||||||||||||||||||||||||||||||||||||||||||
Subject 301  AATAATAATGATGATGAAACTAACCCGAGGGAGCAACTAGCTCCCACTCTGTTGAACTTG  360
Query   499  GCTCTTGAAACCATGCAAAAGATGAGTGAAGACTCGAACATGAGTGTTGTT  549
             |||||||||||||||||||||||||||||||||||||||||||||||||||
Subject 361  GCTCTTGAAACCATGCAAAAGATGAGTGAAGACTCGAACATGAGTGTTGTT  411
```

Supplementary file 10: Figure S2. The supporting alignment result of the coding sequence of *GbCAT7* (GOBAR_AA20422) and Gh_A07G1555. Query, the coding sequence of *GbCAT7*; Subject, the coding sequence of Gh_A07G1555.

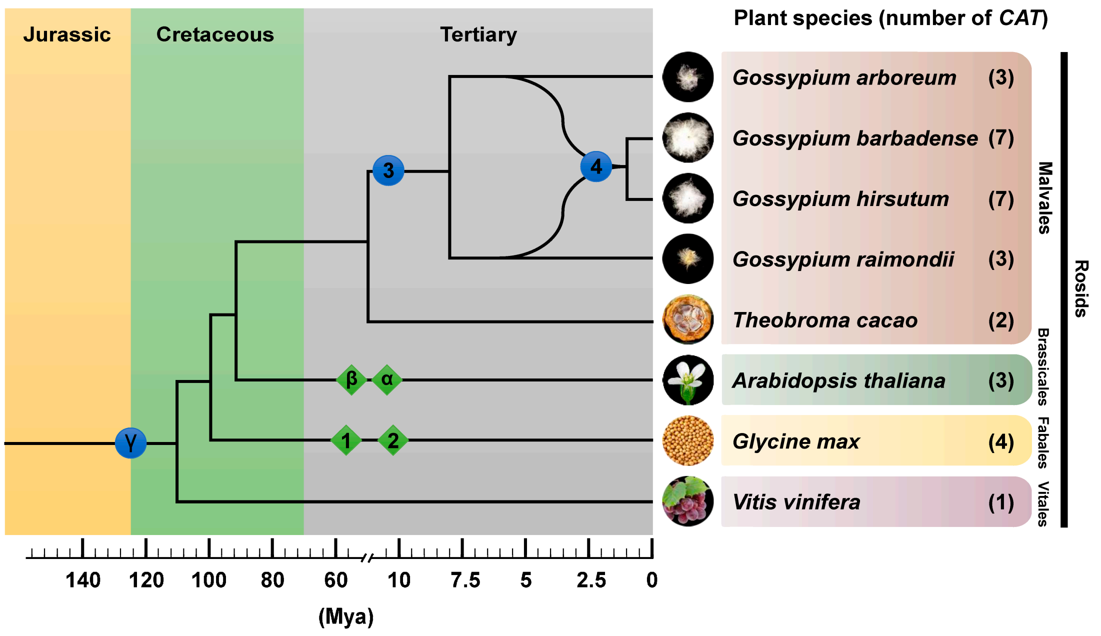

Supplementary file 11: Figure S3. Phylogenetic tree of *Gossypium* and four other genomes descended from common eudicot genome ancestor in Rosids and the number of *CAT* genes identified from the genomes of these plants. The phylogenetic tree of the 8 plants used in the study was gather from the PLAZA database (version 4.0). **WGD**, whole-genome duplication; **Mya**, million years ago; **γ**, one whole genome triplication (WGT) event probably shared by all core eudicots ~130.8 Mya; **α** and **β**, two recent WGDs within the crucifer lineage; **1** and **2**, two Glycine WGD events; **3**, one penta- or hexaploid duplication event in the *Gossypium* lineage after splitting from cacao lineage ~16.6 Mya; **4**, neoallopolyploids reuniting divergent *Gossypium* genomes 1-2 Mya.

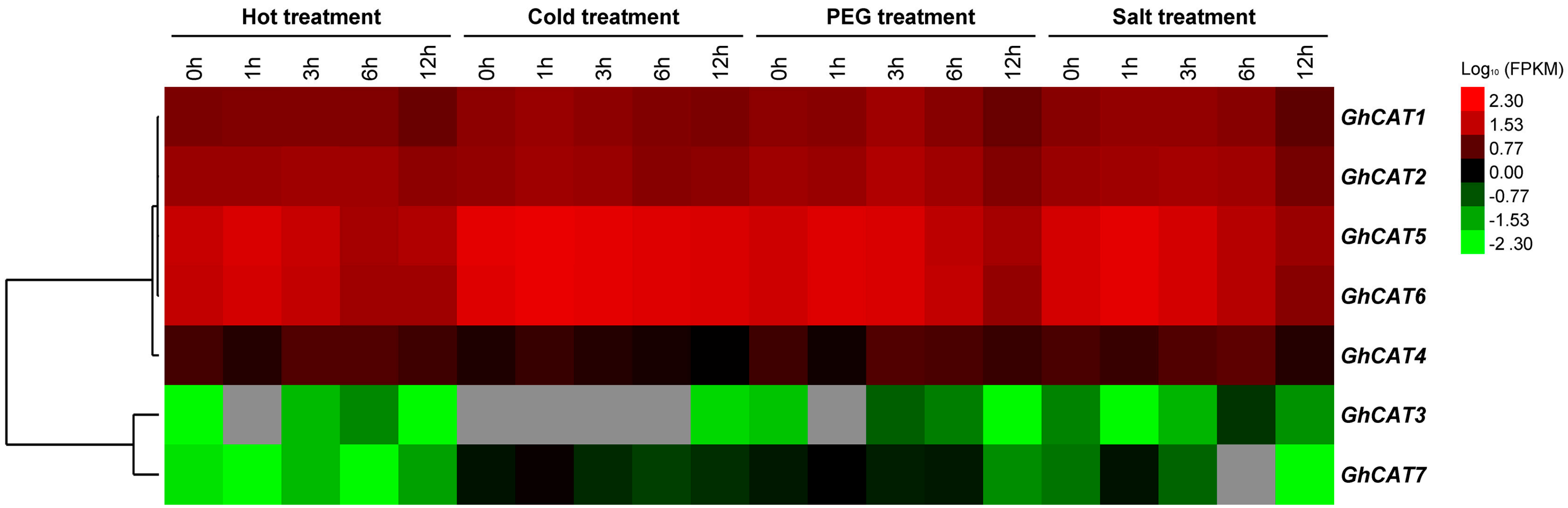

Supplementary file 12: Figure S4. Transcriptional profiling of *GhCATs* under different abiotic stress treatments. The $\log_{10}$ of FPKMs values calculated by RNA-seq data were shown as a heat map. The heat-map showed the hierarchical clustering of the relative expression of 7 *GhCATs* under hot, cold, PEG and salt treatments. The colors of the bar shown to the right of the heat-map varied from red to green representing the relative expression levels from high to low. FPKMs data was obtained from ccNET (http://structuralbiology.cau.edu.cn/gossypium/) and CottonFGD (https://cottonfgd.org/).

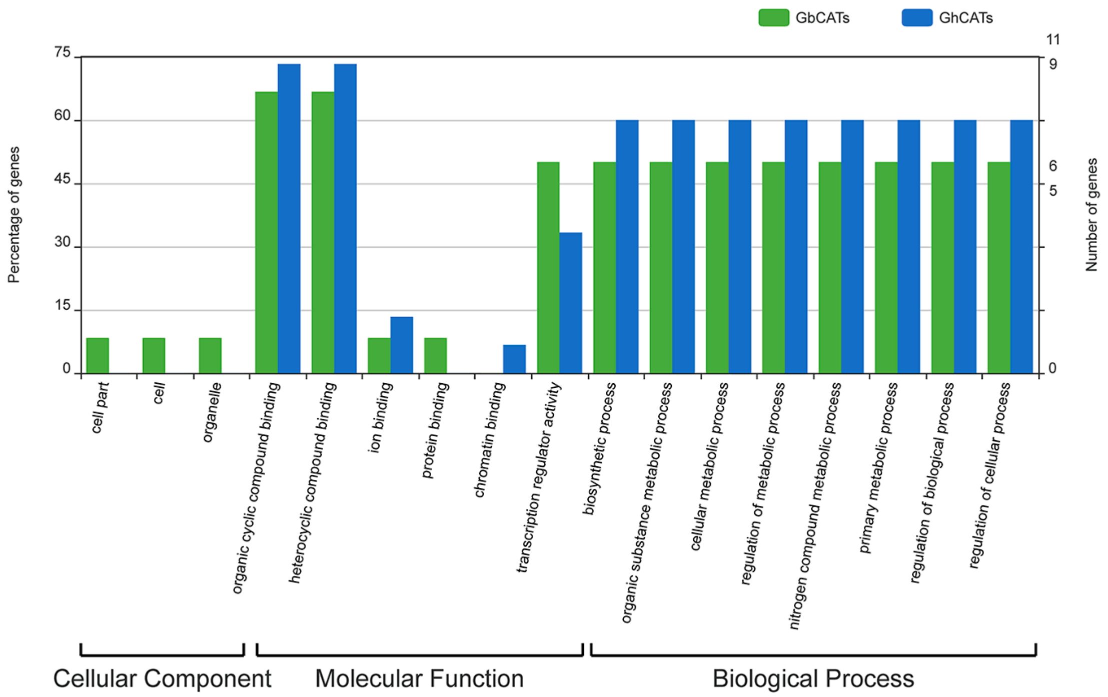

Supplementary file 13: Figure S5. Gene Ontology (GO) annotation of the TFs binding to the upland cotton and sea-island cotton *CAT* genes' putative promoter regions based on their cellular component, molecular function, and biological process. The functional enrichment analysis was carried out by the Gene Ontology Consortium (http://geneontology.org/) and were visualized and plotted by WEGO (version 2.0) (http://wego.genomics.org.cn/).

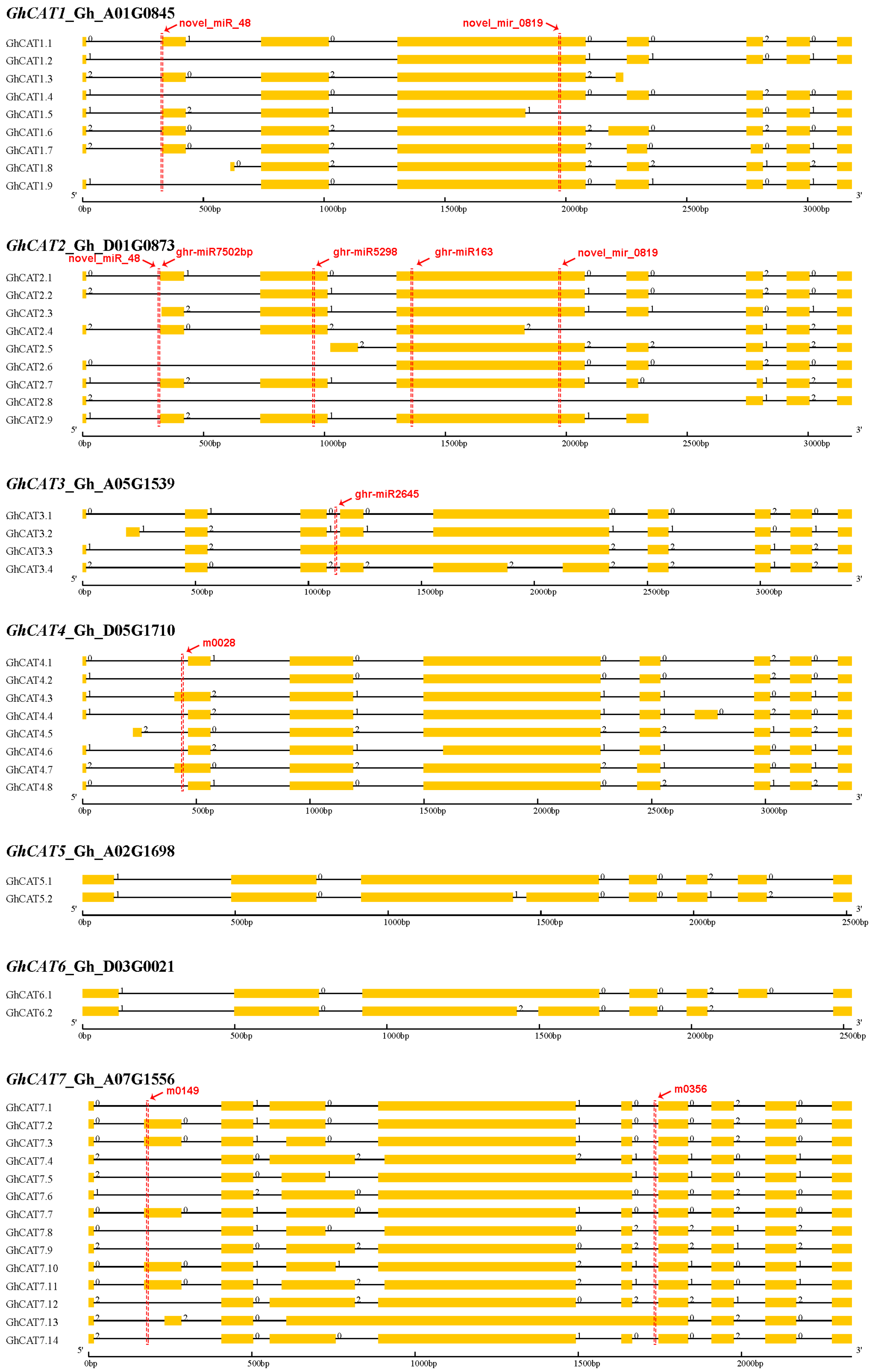
GhCAT1_Gh_A01G0845
novel_miR_48
novel_mir_0819
GhCAT1.1
GhCAT1.2
GhCAT1.3
GhCAT1.4
GhCAT1.5
GhCAT1.6
GhCAT1.7
GhCAT1.8
GhCAT1.9
5'
3'
0bp
500bp
1000bp
1500bp
2000bp
2500bp
3000bp
GhCAT2_Gh_D01G0873
novel_miR_48
ghr-miR7502bp
ghr-miR5298
ghr-miR163
novel_mir_0819
GhCAT2.1
GhCAT2.2
GhCAT2.3
GhCAT2.4
GhCAT2.5
GhCAT2.6
GhCAT2.7
GhCAT2.8
GhCAT2.9
GhCAT3_Gh_A05G1539
ghr-miR2645
GhCAT3.1
GhCAT3.2
GhCAT3.3
GhCAT3.4
GhCAT4_Gh_D05G1710
m0028
GhCAT4.1
GhCAT4.2
GhCAT4.3
GhCAT4.4
GhCAT4.5
GhCAT4.6
GhCAT4.7
GhCAT4.8
GhCAT5_Gh_A02G1698
GhCAT5.1
GhCAT5.2
2500bp
GhCAT6_Gh_D03G0021
GhCAT6.1
GhCAT6.2
GhCAT7_Gh_A07G1556
m0149
m0356
GhCAT7.1
GhCAT7.2
GhCAT7.3
GhCAT7.4
GhCAT7.5
GhCAT7.6
GhCAT7.7
GhCAT7.8
GhCAT7.9
GhCAT7.10
GhCAT7.11
GhCAT7.12
GhCAT7.13
GhCAT7.14

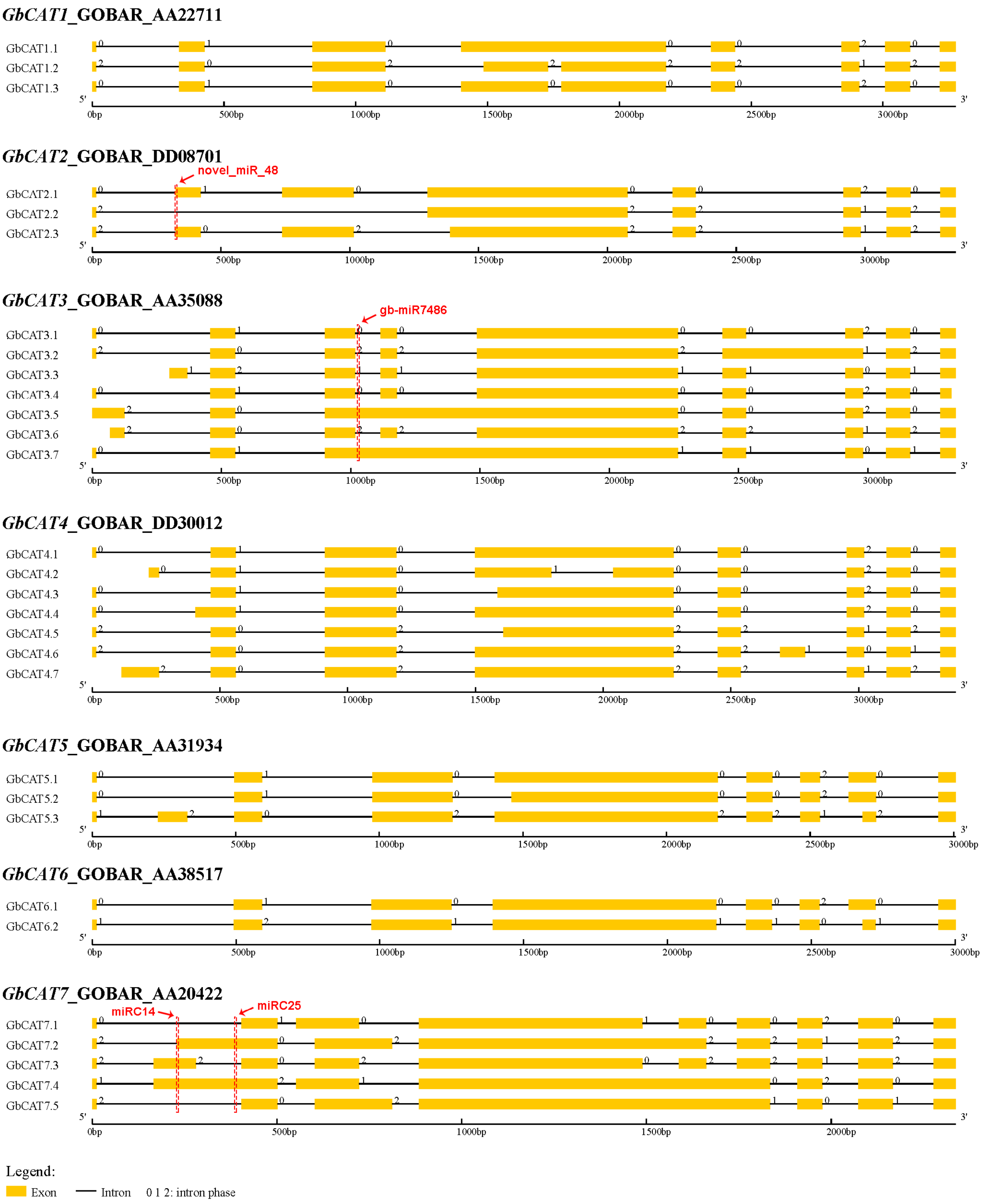

Supplementary file 14: Figure S6. All potentially alternative spliced isoform structure of *GhCATs* and *GbCATs* full-length transcripts and identification of full-length isoforms targeted by cotton miRNAs. The first gene model of each *CAT* gene was the principle transcript, followed these were potential alternative spliced isoforms predicted with mRNA-seq data. The red dotted rectangles were the predicted miRNA targeting sites.

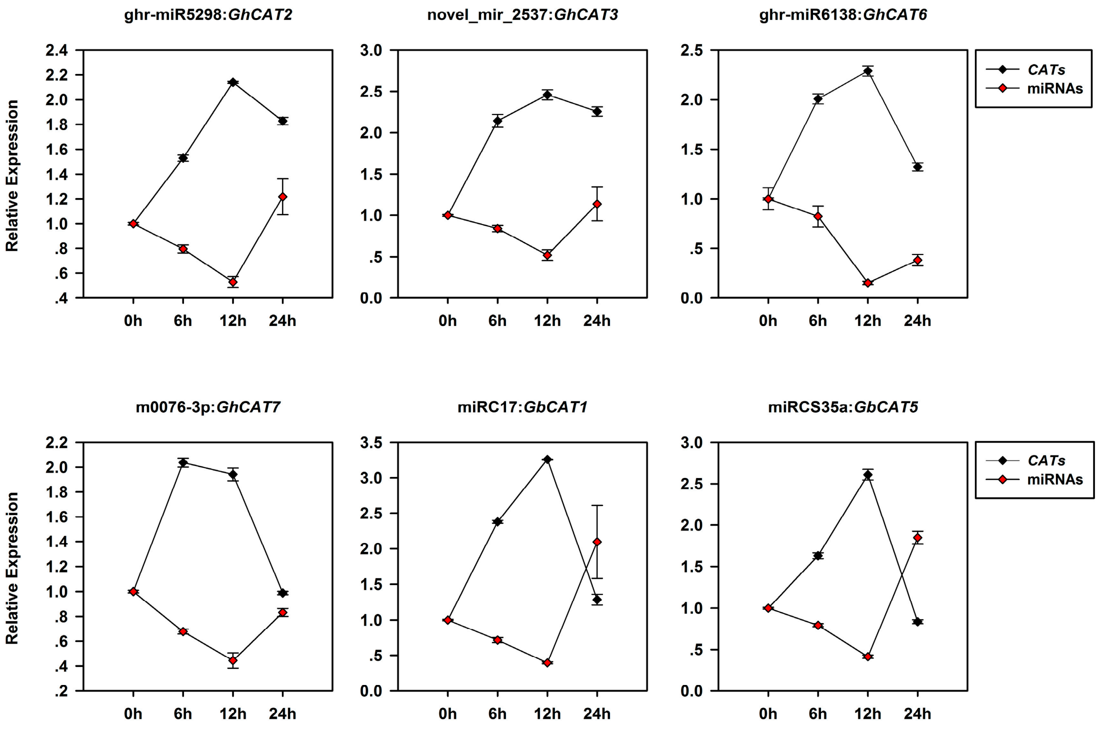

Supplementary file 15: Figure S7. The relative expression levels of miRNAs and their putative targeting *CAT* genes under *V. dahliae* infection treatment. qPCR quantifications were normalized to the expression of *UBQ7* of cotton. Error bars represent standard deviations of mean value from three biological replicates. ANOVA (analysis of variance) was calculated using DPS and $p < 0.05$ was considered statistically significant.

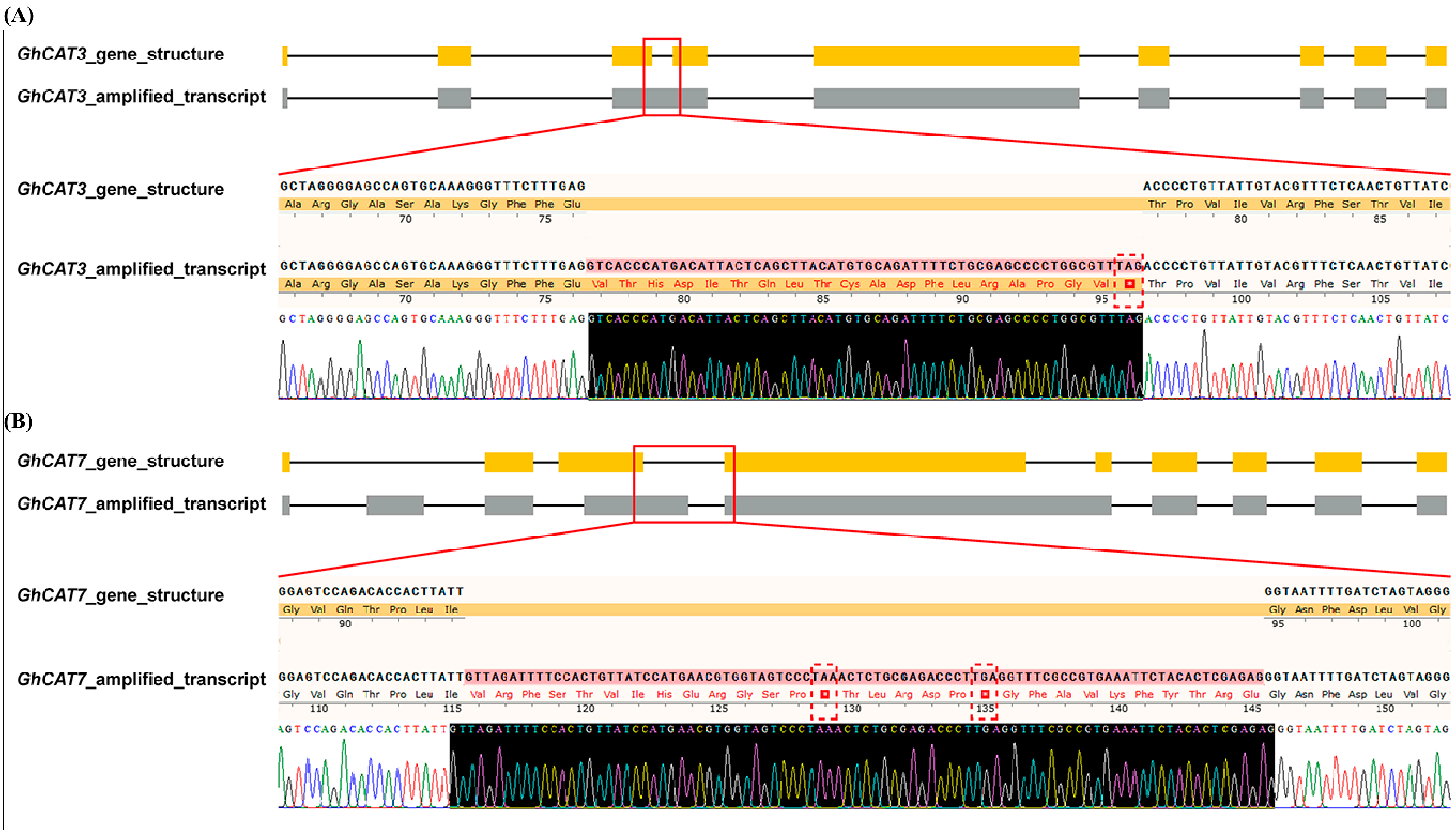

Supplementary file 16: Figure S8. **(A, B)** Amplification of *GhCAT3* (Gh_A05G1539) and *GhCAT7* (Gh_A07G1556) by PCR with the designed primer pairs for cloning their transcripts. The largest-rate transcripts of *GhCAT3* and *GhCAT7* were shown (21/24 and 22/48 respectively in *GhCAT3* and *GhCAT7*). PCR results were aligned with gene structure annotation and were verified by Sanger sequencing. Red dotted rectangle represented the premature termination codons (PTC).
